# Unacylated Ghrelin Rapidly Modulates Lipogenic and Insulin Signaling Pathway Gene Expression in Metabolically Active Tissues of GHSR Deleted Mice

**DOI:** 10.1371/journal.pone.0011749

**Published:** 2010-07-26

**Authors:** Patric J. D. Delhanty, Yuxiang Sun, Jenny A. Visser, Anke van Kerkwijk, Martin Huisman, Wilfred F. J. van IJcken, Sigrid Swagemakers, Roy G. Smith, Axel P. N. Themmen, Aart-Jan van der Lely

**Affiliations:** 1 Department of Internal Medicine, Erasmus MC, Rotterdam, The Netherlands; 2 Center for Biomics, Erasmus MC, Rotterdam, The Netherlands; 3 Center for Bioinformatics, and Department of Genetics, Erasmus MC, Rotterdam, The Netherlands; 4 United States Department of Agriculture/Agricultural Research Service Children's Nutrition Research Center and Huffington Center on Aging, Baylor College of Medicine, Houston, Texas, United States of America; 5 Department of Metabolism and Aging, The Scripps Research Institute Florida, Jupiter, Florida, United States of America; University of Córdoba, Spain

## Abstract

**Background:**

There is increasing evidence that unacylated ghrelin (UAG) improves insulin sensitivity and glucose homeostasis; however, the mechanism for this activity is not fully understood since a UAG receptor has not been discovered.

**Methodology/Principal Findings:**

To assess potential mechanisms of UAG action *in vivo*, we examined rapid effects of UAG on genome-wide expression patterns in fat, muscle and liver of growth hormone secretagogue receptor (GHSR)-ablated mice using microarrays. Expression data were analyzed using Ingenuity Pathways Analysis and Gene Set Enrichment Analysis. Regulation of subsets of these genes was verified by quantitative PCR in an independent experiment. UAG acutely regulated clusters of genes involved in glucose and lipid metabolism in all three tissues, consistent with enhancement of insulin sensitivity.

**Conclusions/Significance:**

Fat, muscle and liver are central to the control of lipid and glucose homeostasis. UAG rapidly modulates the expression of metabolically important genes in these tissues in GHSR-deleted mice indicating a direct, GHSR-independent, action of UAG to improve insulin sensitivity and metabolic profile.

## Introduction

Ghrelin was initially identified as a potent GH secretagogue [1] and is O-octanoylated at Ser3 (acylated ghrelin, AG) by ghrelin O-acyl transferase (GOAT) [2], [3], a posttranslational modification required for its activation of the growth hormone secretagogue receptor (GHSR). However, only 5–20% of circulating ghrelin is acylated, the predominant form being unacylated (UAG) [4]. At first UAG was considered an inactive form of ghrelin, although accumulating evidence indicates that UAG can modulate metabolic activities of the ghrelin system either independently or in opposition to those of AG. Examples of these UAG actions include improvement of pancreatic β-cell function and survival [5], [6], and a beneficial role in cardiovascular function [4], [7]. These activities of UAG would likely have important implications in the progression of the metabolic syndrome. Although evidence exists in these and other systems that UAG acts via a GHSR-independent mechanism, it remains unclear if this is the case for direct regulation of metabolic pathways.

This study aims to address the hypotheses that: a) UAG modulates the expression of genes encoding components of lipid and carbohydrate metabolic pathways, b) UAG acts via a GHSR-independent mechanism. The development of type 2 diabetes (T2D) stems from the suppression of insulin sensitivity in three key organs: the liver, muscle and adipose. The basis for this is deranged lipid metabolism in parallel with altered carbohydrate metabolic pathways. In humans, our published data suggest that UAG can improve insulin sensitivity by modulating lipid metabolism; the co-administration of AG and UAG reduces plasma FFA in GH-deficient patients [8] and the continuous infusion of UAG decreases FFA in healthy and diabetic subjects [9]. This agrees with the findings that fat-specific overexpression of UAG in mice lowers fat mass and improves insulin sensitivity [10], and that UAG has direct effects on adipose tissue *in vitro* [11], [12]. We have also shown that AG and UAG modulate hepatocyte function in which glucose output was stimulated by AG and inhibited by UAG [13]. Like our clinical data, this study showed that UAG counteracts AG stimulated glucose release. The *in vivo* effects of AG and UAG on hepatic insulin sensitivity have been further examined by hyperinsulinemic-euglycemic clamp [14]. It was shown that co-administration of UAG with AG neutralizes the insulin desensitizing effects of AG administration, and normalizes hepatic insulin sensitivity, reinforcing our idea that UAG opposes AG in regulating hepatic glucose metabolism. That the relationship between AG and UAG may have an impact on metabolism has been suggested from clinical studies which show an indirect relationship between circulating AG/UAG ratio and insulin resistance [15], and a decreased AG/UAG ratio in fasting, relatively insulin sensitive, subjects [16].

There is, therefore, indirect evidence that UAG (and AG/UAG ratio) alters lipid and glucose homeostasis *in vivo*, but no direct evidence that it can regulate metabolic pathways that control these processes or of the cellular mechanism(s) involved. To address the hypothesis that UAG can modulate metabolic pathways relevant to insulin sensitivity, we used an unbiased approach: transcriptome-wide expression profiling of liver, muscle and white adipose tissue (WAT). The study examined acute effects of UAG to dissect its direct effects on the tissues examined, including possible signaling pathways. To test the hypothesis that UAG acts independently of the GHSR we examined the effects of UAG on tissues in *Ghsr* knockout mice [17]. Finally, the hypothesis that AG interacts with UAG in regulating metabolic pathways, we examined the effects of UAG on tissues in wild type mice. We find that UAG regulates genes involved in lipid and carbohydrate metabolic pathways in all three tissues in a direction that indicates an overall improvement in metabolic profile, independently of the GHSR.

## Results

### Ingenuity Pathways Analysis (IPA)

**Figure 1:**
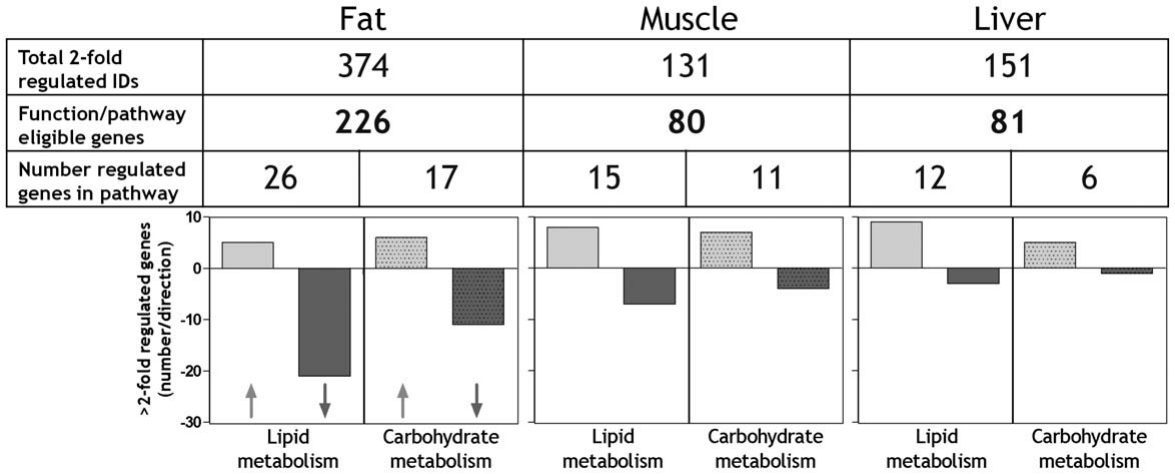
UAG regulates lipid and carbohydrate metabolic pathway genes in fat, muscle and liver of *Ghsr* KO mice. Datasets including two-fold, or greater, regulated genes from each tissue were interrogated by Ingenuity Pathways Analysis for association with all included pathways. Probabilities for random association (false discovery rate) were adjusted using the Benjamini-Hochburg multiple testing correction method. For lipid and carbohydrate metabolic pathways in the fat and liver datasets adjusted *p* was <0.0017, and in the muscle dataset adjusted *p* was <0.0012.

The main finding from the microarray experiment was that UAG caused rapid changes in expression of hundreds of genes in all three tissues examined in adult *Ghsr* knockout mice (Fig. 1). Initially we assessed “regulated” (UAG/wild type ratio ≥2 or ≤0.5) genes in normalized Affymetrix array datasets using Ingenuity Pathways Analysis (IPA, www.ingenuity.com).

Overall, in *Ghsr* deficient mice, UAG modulated by greater than 2-fold the signal from 374 Probe Set IDs in WAT, 131 in muscle and 151 in liver (data corrected using the Benjamini-Hochburg multiple testing method, with effects considered significant at p<0.05). This reflects our findings, and that of others, that UAG influences insulin sensitivity by modulating fat metabolism [10], [18]. After collapsing the IDs into known gene symbols, 226 genes were eligible for pathway analysis in WAT; nearly three times the number that were regulated by 2-fold or more in muscle (80 genes) and liver (81 genes). Of these, several were directly linked with lipid and carbohydrate metabolic pathways (Fig. 1). The majority of genes linked with these two pathways in WAT were down-regulated, whereas in muscle up and down-regulated genes were evenly balanced. In contrast, in liver most genes linked with lipid and carbohydrate metabolism were up-regulated, predominantly in the lipid metabolism pathway.

The data shown in Fig. 1 are based on initial IPA Functional Analyses of the datasets and are derived from the Molecular and Cellular Functions data subset. UAG regulated genes were also significantly linked with other pathways (Table 1). From this analysis it is clear that although UAG-regulated genes show significant associations with lipid and glucose metabolic pathways in all tissues, in muscle and liver these genesets ranked lower in terms of p-value than in WAT. UAG regulated genes in WAT were associated with 24 functions of which lipid and carbohydrate metabolism ranked 4^th^ and 2^nd^, respectively. On the other hand, these functions ranked 8^th^ and 16^th^ of 25 functions in muscle and 11^th^ and 23^rd^ of 27 functions in liver. Many of the top functions were linked with cell growth and proliferation, indicating effects on tissue differentiation or growth.

**Table 1:** Top 5 Ingenuity pathway analysis molecular and cellular functions assigned to ≥2 fold UAG regulated gene sets from fat, muscle and liver of *Ghsr* KO mice.

Fat		
Function	p-value	# Molecules
Cell Signaling	4.37E-09–1.44E-02	80
Carbohydrate Metabolism	1.89E-09–1.44E-02	12
Small Molecule Biochemistry	1.89E-09–1.44E-02	27
Lipid Metabolism	9.86E-09–1.44E-02	19
Nucleic Acid Metabolism	9.86E-09–1.44E-02	5

Muscle		
Function	p-value	# Molecules
Cellular Movement	8.13E-09–1.08E-02	24
Cellular Development	1.38E-09–7.37E-02	30
Cell-to-Cell Signaling and Interaction	2.69E-09–1.07E-02	26
Cell Death	2.89E-09–1.08E-02	33
Cellular Growth and Proliferation	8.58E-09–1.07E-02	30

Liver		
Function	p-value	# Molecules
Cell Signaling	2.72E-09–3.06E-02	44
Cellular Growth and Proliferation	1.73E-09–3.06E-02	29
Cell Death	4.23E-09–3.70E-02	31
Cellular Development	1.21E-09–3.41E-02	31
Cell Morphology	3.76E-09–3.37E-02	18

The p-value was derived using Fisher's Exact test.

In all tissues at least one of the top molecular and cellular functions was related to cell signaling, although no obvious common pathway was regulated. However, expression of genes encoding several G-protein coupled receptors and their regulatory proteins were modulated in WAT, and to a lesser extent in muscle (Table 2).

**Table 2:** G-protein coupled receptors and regulatory proteins regulated by acute UAG treatment (none were regulated ≥2-fold in liver).

Tissue	Probe Set ID	Molecules	Description	UAG/Sal ratio
**Fat**	1415832_at	*Agtr2*	angiotensin II receptor, type 2	0.4
	1425215_at	*Ffar2*	free fatty acid receptor 2	2.1
	1419301_at	*Fzd4*	frizzled homolog 4 (Drosophila)	2.2
	1418379_s_at	*Gpr124*	G protein-coupled receptor (GPCR) 124	0.5
	1427028_at	*Lgr6*	LRR-containing GPCR 6	0.5
	1450286_at	*Npr3*	natriuretic peptide receptor C	3.4
	1440888_at	*Oxtr*	oxytocin receptor	0.3
	1440785_at	*Rxfp1*	relaxin/insulin-like family peptide receptor 1	0.4
	1425701_a_at	*Rgs3*	regulator of G-protein signaling 3	2.0
	1450659_at	*Rgs7*	regulator of G-protein signaling 7	0.5
**Muscle**	1460123_at	*Gpr1*	GPCR 1	2.1
	1457324_at	*Oprs1*	opioid receptor, sigma 1	2.3
	1440888_at	*Oxtr*	oxytocin receptor	0.4

**Figure 2:**
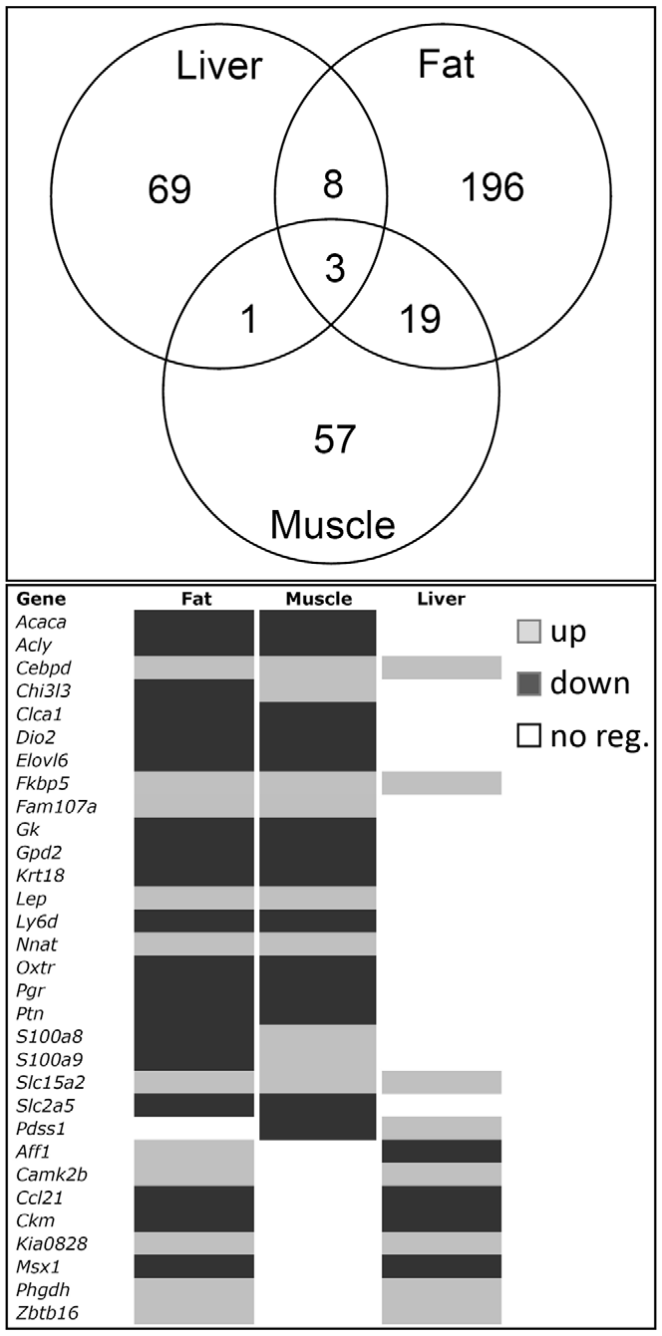
Overlap in the expression of UAG regulated genes amongst tissues of *Ghsr* KO mice. The largest intersecting set occurs between fat and muscle with 22 genes (Venn diagram). These genes tend to be regulated in the same direction in both tissues (18 of 21, heat map on right) and include genes that encode key regulatory enzymes in lipid and carbohydrate metabolism, such as *Acaca* and *Acly*. Intersects between fat and liver, and muscle and liver consist of much smaller gene sets, but include *Camk2b*, *Ckm*, *Phgdh* and *Zbtb16*, which have all been linked with regulation of energy homeostasis and metabolic syndrome.

Having established that, in the absence of the GHSR, UAG regulates gene expression in WAT, muscle and liver, we asked if the pattern of gene expression amongst these tissues was similar. This could suggest commonalities in UAG's mechanism of action. Of the total of 353 genes, less than 1% are regulated by UAG in all three tissues (Fig. 2). Interestingly, one of these 3 genes was *Cebpd*. CAAT enhancer binding protein δ (C/EBPδ) is an important initiator of adipocyte differentiation, and is involved in myocyte and hepatocyte differentiation. In an independent experiment *Cebpd* was confirmed to be significantly suppressed by UAG in WAT and muscle, and showed a trend to increase in liver. *Fkbp5* (FK506 BP5) and *Slc15a2* (proton/oligopeptide transporter) are also regulated by UAG in all three tissues, although their function in these settings is unclear. The greatest overlap of UAG regulated genes occurred between WAT and muscle (Fig. 2), particularly those encoding enzymes involved in fatty acid synthesis (*Acaca*, *Acly, Gyk* and *Elovl6*), glucose transport (*Slc2a5*), the ox-phos pathway (*Gpd2*), and adipogenesis (*Cebpd*, *Lep*). Importantly, these genes tended to be down-regulated in both WAT and muscle.

### Gene Set Enrichment Analysis (GSEA)

Gene Set Enrichment Analysis (GSEA) was used to analyze the entire microarray data set from each tissue [19].

#### White adipose tissue

In WAT, UAG upregulated gene sets for N-glycan degradation, fibrinolysis/complement pathways, the CD40 pathway, the Akt pathway, and the glutathione metabolic pathway (Table S1). UAG down-regulated gene sets (Table S2) relating to fatty acid, cholesterol/steroid and glucose/carbohydrate metabolism, and mitochondrial respiration (OXPHOS and Krebs/TCA cycle pathways), as well as gene sets linked with T2D and Maturity Onset Diabetes of the Young (MODY), and insulin signaling.

GSEA was also used to interrogate gene sets that contain specific *cis*-regulatory elements (Tables S3 & S4). Transcripts of genes containing E2F, ATF2 and REST *cis*-elements were upregulated by UAG in *Ghrs* KO WAT. Transcripts of genes containing, for example, SRF, AP2, CEBP, and FOX *cis*-elements were downregulated by UAG.

#### Muscle

In muscle, UAG upregulated gene sets that were predominantly associated with myocyte (RARALPHA, MYOD_NIH3T3) and adipocyte (IDX_TSA, NADLER_OBESITY_UP) differentiation, as well as WNT, IGF-I and insulin signaling (Table S5). Down-regulated gene sets in muscle include the pentose phosphate pathway, as well as pathways involved with lipid and carbohydrate metabolism (Table S6).

Assessment of transcript gene sets containing specific *cis*-regulatory elements that were upregulated by UAG in muscle are detailed in Table S7. These include genes containing E2F, C/EBP(β/δ), CREB, SREBP, GATA, MYOGENIN and MYOD *cis*-elements. Transcripts of genes containing HNF1 *cis*-elements were downregulated by UAG.

#### Liver

UAG upregulated gene sets in liver, as in fat and muscle, included those related to hepatocyte growth and adipogenesis (eg. LEE_MYC_E2F1_UP and IDX_TSA_UP_CLUSTER1; Table S8). Additionally, three gene sets related to mitochondrial oxidative respiration (HAS00190_OXIDATIVE_PHOSPHORYLATION, CITRATE_CYCLE_TCA_CYCLE and ELECTRON_TRANSPORT_CHAIN) were upregulated. Assessment of transcript gene sets containing specific *cis*-regulatory elements that were upregulated by UAG in liver (Table S9) include ATF, CREBP1 and RSRF4.

### Quantitative PCR gene expression confirmation

We next validated the microarray data analyses in an independent experiment. Six *Ghsr* knockout mice were injected with either saline or UAG and 6 hours later tissues were removed for analysis of gene expression by quantitative PCR. We assessed genes identified in the initial expression profiling experiment to be either regulated more than 2-fold by UAG treatment, or those linked with UAG-regulated pathways identified by GSEA, particularly lipid and carbohydrate metabolism, and adipogenesis. Between the two independent (microarray and QPCR) experiments, approximately 70% (15) of the genes assessed show strong correlation (*r^2^* = 0.8, *p*<0.0001) in direction and magnitude of regulation by UAG (Figure S1).

#### White adipose tissue

**Figure 3:**
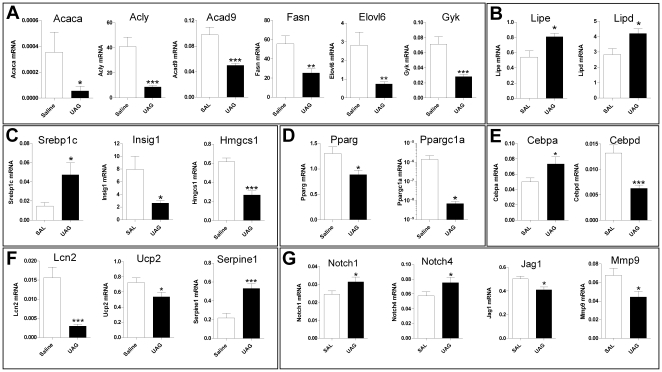
Confirmation of UAG regulated genes in white adipose tissue. Quantitative PCR measurement of gene expression in an independent experiment confirms regulation by UAG of genes involved in lipid/cholesterol metabolism, as well as adipocyte differentiation and insulin-sensitivity in white adipose tissue (WAT). A: Key regulators of fatty acid synthesis, both short and long-chain, were suppressed by UAG treatment. B: Hormone sensitive lipase (*Lipe*) and liporotein lipase (*Lipd*) gene expression is increased. C: Regulators of cholesterol synthesis, *Insig1* and *Hmgcs1* are suppressed by UAG, whereas *Srebp1c* is induced. D: Expression of *Pparg* and its coactivator *Ppargc1a*, mRNAs that encode key regulators of lipid synthesis were suppressed by UAG. *Pparg* is also a key regulator of adipocyte differentiation. E: Key transcriptional regulators of adipocyte differentiation, *Cebpa* and *Cebpd* are up and down-regulated, respectively, by UAG in fat, suggesting suppressive effects on the early stages of differentiation. F: Regulation of *Serpine1*, *Lcn2* and *Ucp2* by UAG indicates improvement of insulin sensitivity in WAT. G: Components of the Notch signalling (*Notch1/4*, *Jagged1*) and the fibrinolytic (*Mmp9*) pathways (inhibitory and permissive for adipocyte differentiation, respectively) are regulated by UAG.

In gonadal WAT many genes linked with lipogenesis were significantly down-regulated (Fig. 3). These include *Acaca* (acetyl CoA carboxylase α), *Acly* (ATP citrate lyase), *Acad9* (acetyl CoA dehydrogenase 9), *Fasn* (fatty acid synthase), *Elovl6* (ELOVL family member 6, elongation of long chain fatty acids) and *Gyk* (glycerol kinase) (Fig. 3A). Hormone sensitive lipase and lipoprotein lipase mRNAs (*Lipe* and *Lipd*) were significantly increased (Fig. 3B). Genes that control sterol/cholesterol synthesis are also regulated. For example, *Hmgcs1* (cytosolic hydroxymethylglutaryl CoA synthase) and *Insig1* (insulin induced gene 1) mRNAs are suppressed and *Srebp1c* (sterol response-element binding protein 1c) is induced (Fig. 3C). There is recent evidence that SREBP1c, in contrast to its function in liver, regulates cholesterol synthesis not lipogenesis in fat [20], [21]. In fat there appears to be uncoupling of this transcription factor from regulation of genes such as *Acaca* and *Fas*, which could explain our finding that these genes are not coordinated with *Srebp1c* expression. We found in the array data that *Srebp2* expression is suppressed by approximately 30%, a possible mechanism for the down-regulation of lipogenic genes, although this transcription factor preferentially regulates cholesterologenesis [22]. Further work needs to be performed to determine the transcriptional mechanisms involved. *Insig1* is also a target gene for PPARγ activation, and its down-regulation may be linked with the down-regulation of both *Pparg* and *Ppargc1a* (Fig. 3D).

Down-regulation of the key regulator of adipogenesis, *Pparg*, indicated modulation of differentiated state in adipose tissue, as revealed by GSEA analysis. Additionally, GSEA showed that UAG altered the expression of genes containing *cis*-elements that bind adipogenic transcription factors (eg. E2F, p27, p53, SRF [23]). This was borne out by the finding that several genes that either regulate, or are markers for, adipogenesis were altered acutely by UAG treatment. For example, mRNAs encoding C/EBPα and C/EBPδ were rapidly up- and down-regulated, respectively, by UAG treatment (Fig. 3E). *Lcn2* (lipocalin 2) and *Ucp2* (uncoupling protein 2) were markedly suppressed by UAG, indicating improved insulin signaling. *Serpine1* (encoding PAI-1) was increased (Fig. 3F). Based on GSEA, we also measured expression of genes involved in Notch signaling (*Notch1/4* and *Jag1*) and matrix remodeling/fibrinolysis (*Mmp9* and *Timp4*) and found them to be regulated by UAG (Fig. 3G). These pathways are important for normal adipogenesis and are altered in obesity [24], [25].

#### Muscle

**Figure 4:**
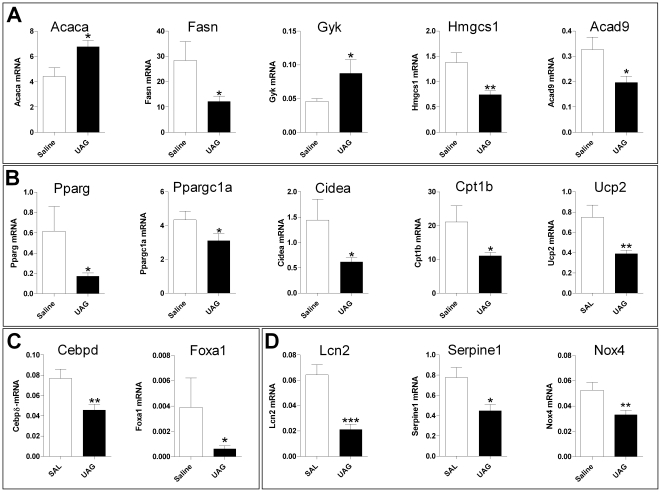
Quantitative PCR measurement of UAG regulated genes in muscle, in an independent experiment. A: Genes encoding components of fatty acid, triglyceride and cholesterol synthetic pathways. B: Genes encoding key regulators of lipid metabolism and lipid handling. C: Genes encoding muscle differentiation. D: Genes encoding modulators of insulin sensitivity and signaling.

UAG significantly regulated a number of genes in muscle that were involved in lipid and carbohydrate metabolism. Like WAT, the majority of these were downregulated, with a few exceptions, including *Acaca* and *Gyk*, which were significantly upregulated (Fig. 4A). The key regulator of these processes, *Pparg* was suppressed, as well as genes linked with mitochondrial respiration, such as *Ppargc1a* and *Cpt1b* (Fig. 4B). Surprisingly, in *Ghsr* KO muscle *Acaca* was expressed at similar levels to *Acacb* (which encodes the predominant form of muscle acetyl CoA carboxylase, ACC2). However, this gene was not regulated by UAG treatment. Two genes that are important in the early regulation of adipogenesis, *Cebpd* and *Foxa1*, are also modulated by UAG (Fig. 4C). Finally, four genes linked with modulation of insulin sensitivity, *Lcn2*, *Ucp2*, *Serpine1* and *Nox4*, are suppressed by UAG treatment in muscle (Fig. 4D).

#### Liver

**Figure 5:**
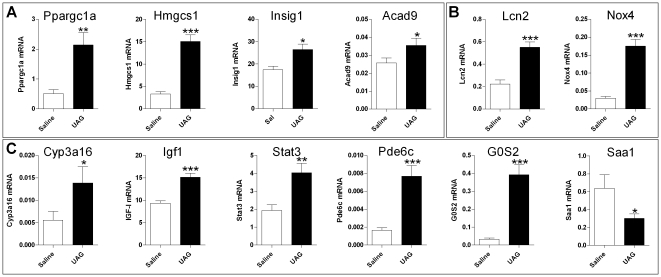
Quantitative PCR measurement of UAG regulated genes in liver, in an independent experiment. A: Genes encoding components of lipid and cholesterol synthetic pathways. B: Genes encoding modulators of insulin sensitivity and signaling. C: Genes encoding markers of GH responsiveness and components of intra-cellular signaling pathways.

Liver responded differently from WAT and muscle in that genes encoding components of lipid and carbohydrate metabolic pathways, if they were regulated at all, were upregulated (Fig. 5A). This fits with GSEA analyses, where adipogenic pathway gene sets were upregulated in liver as opposed to mostly being down-regulated in WAT and muscle. Conversely, *Lcn2* and *Nox4*, genes linked with insulin sensitivity that were downregulated in WAT and muscle, were upregulated (Fig. 5B). *Saa1*, a marker of high fat diet induced hepatic insulin resistance, is suppressed by UAG. Lastly, it was found that several upregulated genes indicated the influence of UAG on signaling pathways involving GH (*Cyp3a16*, *Igf1*), cytokines (*Stat3*), cAMP (*Pde6c*) and PPARγ (*G0S2*) (Fig. 5C).

## Discussion

The goal of this study is to identify in an objective way the effects of UAG on gene expression in metabolically important tissues in order to extract information about the cellular mechanism for UAG function in the absence of the GHSR. We find that UAG generally down-regulates genes that encode components of lipid and carbohydrate metabolic pathways in WAT and muscle and up-regulates them in liver, indicating an overall improvement in metabolic profile. This corroborates earlier clinical and animal studies showing that UAG can improve lipid and glucose homeostasis, and demonstrate that UAG has functional effects on pathways that regulate lipid and glucose metabolism. Agreement between the independent microarray and QPCR data strengthen this conclusion. Even more interesting is the discovery that UAG upregulates pathways involved in insulin signaling. This suggests direct regulation of insulin sensitivity, particularly in WAT, as well as cross-talk between UAG and insulin signaling pathways. Collectively, these effects of UAG are similar to the finding that partial antagonism, or reduced expression, of PPARγ blocks its obesogenic effects, but maintains the ability of PPARγ to improve insulin sensitivity [26], [27].

The second important finding is that peripherally administered UAG has activity in the absence of the GHSR, as indicated in pathways analyses by the modulation of diverse signaling pathways including [Ca^2+^]_i_, cAMP, “growth factors”, Notch and Wnt. Furthermore, the gene expression of a number of G-protein coupled receptors (GPCR) and their regulatory proteins were modulated by UAG in WAT and muscle. Such candidate receptors may be regulated via feedback mechanisms by their ligands and it is possible that one of them is responding to UAG treatment. The only GPCR regulated by UAG in both WAT and muscle was the oxytocin receptor. Oxytocin has been shown to modulate lipogenesis and glycolysis in fat [28], and more recently to have metabolic function in skeletal myoblasts and cardiomyocytes [29], [30]. However, we have demonstrated *in vitro* that this receptor does not signal for UAG (data not shown). Of course, the UAG receptor could be another class of receptor, such as a tyrosine kinase receptor. Although no obviously consistent pattern that could define a UAG signaling pathway was determined, it was found that there is overlap in the specific genes that are regulated by UAG in the different tissues, particularly between WAT and muscle. Importantly, the majority of these genes are co-regulated in the same direction across tissues pointing to commonality in the mechanism of UAG action.

### White adipose tissue

#### Lipid metabolism

The main finding of this study is that UAG suppresses genes that encode key regulatory enzymes involved in lipogenesis and sterol synthesis in WAT. In relation to this finding, micro-array data also show >2-fold suppression of *Lipg* (endothelial lipase) that mediates uptake of HDL particles and promotes ApoAI mediated cholesterol efflux [31]. In humans there is a direct correlation between LIPG levels and BMI and waist circumference [32]. *Insig1* is a target gene for PPARγ activation, and its down-regulation may be linked with the down-regulation of both *Pparg* and *Ppargc1a*. Although UAG treatment of *Ghsr* deficient mice primarily affects lipogenesis, we considered that the lipolytic pathway may also be modulated, since UAG affects lipolysis *in vitro* [12]. Because of the high turnover rate of triacyl glycerol (TAG), an imbalance between the synthesis and hydrolysis of TAG could lead to the development of obesity [33]. We found not only that *Gyk* (glycerol kinase) gene expression was suppressed, but also that the lipases *Lipe* and *Lipd* were moderately, but significantly, induced by UAG treatment. Furthermore, UAG treatment caused a trend towards decreased levels of hepatic TAG after only 6 h (Figure S2).

An unexpected outcome of our analyses was that UAG increases gene-sets involved in N-glycan degradation in WAT. Glycosylation is important for GLUT1, GLUT4 and LPL function, and serum N-linked glycoproteins are increased in obese diabetic mice and humans [34].

#### Insulin sensitivity

Upregulation of the Akt/PKB GSEA geneset is significant since it indicates that the metabolic signalling, but not the cell proliferation, response to insulin is induced. It has recently been demonstrated that this pathway is required for insulin mediated regulation of lipid metabolism in adipocytes [35]. Interestingly, the GSEA insulin signalling geneset (HSA04910_INSULIN_SIGNALING_PATHWAY) was suppressed by UAG in WAT. This geneset consists predominantly of genes encoding participants in the mitogenic IRS-ERK cascade, suggesting that UAG potentiates the metabolic effects of insulin at the expense of its proliferative effects.

*Lcn2* (lipocalin 2), recently implicated in the development of obesity and insulin resistance [36], [37], was markedly suppressed by UAG treatment. *S*uppression of *Lcn2* improves insulin sensitivity of adipocytes in culture and suppresses *Pparg* gene expression [37]. The corollary of this is that suppression of *Lcn2* by UAG improves insulin sensitivity in fat, and suppresses adipogenesis. Likewise, *Ucp2* was suppressed by UAG in adipose tissue. Suppression of the *Ucp2* gene *in vivo* causes increased insulin sensitivity in adipose tissue [38]. The increase in *Serpine1* (encoding PAI-1) seems at odds with the beneficial effects of UAG on lipid and glucose metabolic pathways. Although PAI-1 levels correlate with adiposity in obesity, it seems to have no functional role in adipogenesis [39]. Moreover, transgenic over-expression of *Serpine1* in mice attenuates diet induced obesity. Recent evidence suggests that PAI-1 is up-regulated by insulin [40], therefore the increase we observe may reflect acute improvements in insulin sensitivity in fat.

#### Adipogenesis

The down-regulation of *Pparg* also indicates the modulation of markers of the differentiated state in adipose tissue, as indicated by the GSEA analyses. This was borne out by the finding that several genes that either regulate, or are markers for, adipogenesis were altered by UAG treatment (Fig. 4B). An outcome of the GSEA was the upregulation of Notch receptors by UAG, suggesting an additional inhibitory effect on adipogenesis via this pathway. Another process that is central to the development of fat is tissue remodeling, identified in both Ingenuity Pathways analyses and GSEA (fibrinolytic pathway including matrix metallo-proteases (MMPs)) to be modified by UAG. One of the key MMPs involved in adipogenesis is MMP9, which is down-regulated by insulin and, perhaps due to insulin resistance, raised in obesity [eg.25]. *Mmp9* is suppressed by UAG treatment strongly suggesting an impact on tissue remodeling in WAT.

Our data clearly fit with those of Zhang *et al.* [10], who show that overexpression of UAG in fat cells *in vivo* suppresses adipogenesis and fat accumulation. We now show that these effects are the consequence of a direct response of adipocytes to UAG in the absence of the GHSR *in vivo*.

The overall finding in WAT is that UAG suppresses genes involved in adipogenesis and lipogenesis. This contrasts with AG which causes the accumulation of lipid in WAT by favoring expression of lipogenic genes, or altering lipid handling [41], [42]. Our findings reinforce the idea that UAG counteracts the effects of AG on these metabolic pathways particularly in WAT. Importantly, UAG decreases lipogenesis in fat at the same time as improving insulin sensitivity.

### Muscle

#### Lipid metabolism

In muscle, both pathway and gene-set enrichment analyses show effects of UAG treatment on gene clusters involving adipogenesis. Based on GSEA analyses and QPCR data UAG also suppresses lipid, sterol and carbohydrate metabolism-related gene expression. Finally, UAG treatment was found to suppress gene sets linked with the pentose phosphate (PP) cycle (Table S6). A major role for the PP pathway is to supply NADPH for fatty acid synthesis. Thus, inhibition of this pathway in muscle correlates well with the general suppression of genes that encode lipogenetic pathways. The mechanism of action of UAG on this pathway, potentially through regulation of hexose-6-phosphate dehydrogenase (H6PD), remains to be determined, although H6PD ablated mice have increased insulin sensitivity in glycolytic muscle [43], such as the *vastus lateralis* from which our data are derived.

#### Insulin sensitivity

UAG suppressed *Cidea* gene expression in muscle, although little is known about its function at this site [44]. Cidea, like perilipin and adipophilin, localizes at the surfaces of lipid droplets in adipocytes [45]. Mice lacking *Cidea* are resistant to diet-induced obesity and diabetes through modulation of lipid handling in their tissues [46]. Down-regulation of Cidea suggests improved insulin sensitivity in muscle, corresponding with up-regulation of the AKTPATHWAY GSEA geneset (Table S5), and suppression of *Lcn2* and *Serpine1* that are linked with worsened insulin sensitivity [36], [37], [38].

#### Adipogenesis

Skeletal muscle contains stem cells, or satellite cells, that retain broad differentiation capacity including the ability to generate adipocytes, and myogenic cell lines (eg. C2C12) can be converted to adipocytes by overexpression of PPARγ and C/EBP. Substitution of muscle with fat strongly correlates with insulin resistance. Moreover, hyperglycemia *in vivo* and high-glucose concentrations *in vitro* induce *de novo* lipogenesis and intracellular lipid accumulation in muscle cells [47]. UAG appears to counteract these effects by suppressing genes that stimulate adipogenesis and lipid accumulation. Interestingly, *Cebpd* and *Foxa1* (encoding HNF3α) were down-regulated. Products of these genes, as well as *Pparg* which was also suppressed, are involved in the early stages of adipogenesis [48], and could indicate suppression of adipogenesis in muscle. This is a particularly intriguing finding since it has been shown that the insulin resistance of morbid obesity can be reversed by intramyocellular fat depletion [49].

### Liver

UAG has very little effect on lipogenic pathways in liver, but upregulates oxidative phosphorylation (GSEA analyses, Table S8) and lipid β-oxidation, also indicated by the up-regulation of *Ppargc1a* and *Acad9* (Fig. 5a). ACAD9 deficiency in humans can lead to acute liver dysfunction and hypoglycemia, and deletion of medium chain acyl-CoA dehydrogenase (*Acadm*), immediately downstream of Acad9 in the mitochondrial long-chain β-oxidation pathway, profoundly affects hepatic glucose metabolism [50]. In contrast, AG treatment up-regulates lipid metabolism genes, including *Acaca* and *Fasn* (and carbohydrate metabolism - *G6pc*), and suppresses lipid oxidation as demonstrated by suppression of *Cpt1* [41].

### Conclusion

Collectively, our data show that UAG suppresses genes involved in lipid metabolism, particularly those involved in lipogenesis, in WAT and muscle. Moreover, the combined effect of UAG is to enrich indicators of insulin sensitivity in these tissues, in line with previous clinical and animal studies. Future studies will be directed to assessing both acute and longer-term effects of UAG on these specific processes and pathways not only *in vivo* but also *in vitro*. The *in vitro* work in particular is an important approach to assess our hypothesis that UAG is having direct effects on peripheral tissues. Overall, the current study suggests direct action of peripheral UAG because of the rapidity of its effect on the tissues we have examined. Moreover, we have found that our *Ghsr* KO mice show no modulation of feeding behavior following peripheral (intraperitoneal) injection of UAG [51]. It was only upon intra-cerebroventricular administration that activation of neurons in the lateral hypothalamic area was induced. Therefore, based on findings in our *Ghsr* KO mice we would favour a purely direct action on peripheral tissues by peripherally administered UAG. However, in ddY mice, peripherally administered UAG was shown to stimulate neurons in the hypothalamus, and modulate food intake [18], although it is not clear in these studies if there is a causal link. Interestingly, recent studies in rats showed that peripheral UAG inhibits AG stimulated food intake and hypothalamic neuron activation [52], but unlike the earlier study UAG had no independent effect. This relates more closely with our findings that suggest that UAG does not independently activate a hypothalamic or central pathway, at least in *Ghsr* deficient mice. This is unlike AG, for which regulation of lipid metabolism has been established to occur via a hypothalamic-relay involving the sympathetic nervous system [53], [54], [55], [56]. The model that we have described in this study cannot easily distinguish between a rapid central and a direct effect of UAG, and therefore further work is required to dissect a possible central mechanism of action.

Although the focus of this study was to determine UAG-dependent effects on metabolic processes, our findings also have implications for the interaction of UAG with acylated ghrelin. Findings of our group and others indicate that UAG can act in opposition to the effects of AG. For example, recent studies performed in mice have shown that, in contrast to AG, centrally or intraperitoneally administered UAG induces a negative energy balance by decreasing food intake and delaying gastric emptying [18]. Consistent with these results, peripherally injected UAG blocks the orexigenic effects of AG in rats [52] and transgenic mice that overexpress UAG in fat had improved insulin sensitivity and reduced fat mass [10]. In humans, our data also suggest effects on lipid metabolism; the co-administration of AG and UAG reduces plasma FFA in GHD patients [8] and the continuous infusion of UAG [9] decreases FFA in healthy and diabetic subjects, respectively. The main site of effect amongst the three tissues examined in *Ghsr* KO mice was found to be WAT. The effects of UAG, which may favor decreased adiposity through GHSR-independent suppression of lipogenetic genes, is in opposition to the effects of AG to promote fat accumulation through GHSR-dependent lipid retention [42]. Acylated ghrelin had no effect on lipogenic genes in white adipose [42], and the apparent difference in mechanisms of action of UAG and AG on regulation of adiposity is particularly intriguing, lending support for the presence of a separate UAG receptor.

## Materials and Methods

### Animals

All animal protocols used were approved by Baylor College of Medicine Animal Care and Use Committee (Protocol AN-2770). Twelve week old female *Ghsr*−/− mice (>99.9% congenic with C57BL/6; N12) were kept under conditions of 12∶12 h dark∶light, constant temperature, and provided chow and water *ad libitum*. Murine UAG (NeoMPS, Strasbourg, France) dissolved in saline was injected *ip.* at 20 nmol/kg (200 µl). Controls were injected with 200 µl of saline. Injections were performed between 3 and 4 hours after lights-on. Mice were provided chow and water *ad libitum* until the time of tissue collection. Six hours later animals were euthanized, and tissues were immediately flash-frozen in liquid nitrogen. All samples were stored at −80°C until being processed.

### Microarray analysis

RNA was isolated from gonadal white adipose tissue (WAT), muscle (*M. vastus lateralis*) and liver of saline and UAG treated *Ghsr*−/− mice (n = 2; mean body weight 20.5±0.1 g) and assessed for integrity (RNA Integrity Number (RIN) ≥8.0) on an Agilent 2100 Bioanalyzer (Agilent Technologies, Waldbronn, Germany)[57]. RNA was then processed for hybridization on mouse Genome 430 2.0 Affymetrix microarrays at the Erasmus Center for Biomics, using standard Affymetrix protocols. The mean number of present calls on the arrays was 55.6±0.6%, and the β-actin and GAPDH 3′/5′ ratios were within normal ranges. The normalized array data have been deposited at the Gene Expression Omnibus archive, accession number GSE22506.

### Real-time quantitative PCR

In a separate experiment, groups of 6 mice (mean body weight 19.9±0.4 g) were treated in an identical procedure to that described above. RNA was isolated, and 0.8 µg RNA was reverse transcribed using M-MLV RT (Promega, The Netherlands) and an oligo-dT/random hexamer priming mix (Roche, The Netherlands). QPCR was performed using a qPCR Core kit for SYBR Green I (Eurogentec, The Netherlands). Gene-specific primers were designed to span introns, and data were corrected for β-actin gene expression (primer sequences available upon request). Gene expression data derived from quantitative PCR experiments were analyzed by Student's t-test, with effects being considered significant at p<0.05.

### Statistical analyses

Array intensities and calls were collected using R, and quantile normalization was used on one time present calls. An intensity threshold of 30 was then applied to generate datasets for analysis. Two-fold regulated genes (≥2 or ≤0.5, calculated as the ratio of the means) were analyzed using Ingenuity Pathways Analysis (Ingenuity Systems, www.ingenuity.com). Probabilities for random association (false discovery rate, FDR) were adjusted using the Benjamini-Hochburg multiple testing correction method, with effects being considered significant at p<0.05.

Gene Set Enrichment Analysis software (v. 2.0.4) was used to analyze the entire data set from each tissue. These data were initially expressed as log2 ratios of the means of the control and UAG treated experimental groups. We then used GSEA to interrogate two *a priori* defined molecular signature databases at the Broad Institute (www.broad.mit.edu/gsea/msigdb/index.jsp): a manually curated pathway database (c2.cp.v2.5.symbols) and a transcription factor targets database (c3.tft.v2.5.symbols.gmt) [19]. Data are presented if the false discovery rate (FDR) *q*-value for the gene set is less than 0.25.

## Supporting Information

Figure S1 Approximately 70% of genes assessed by QPCR in fat from the independent Experiment 2 correlated strongly, in terms of direction and magnitude of regulation by UAG, with the array data derived from Experiment 1 (r2, 0.8; p<0.0001).
(0.07 MB TIF)

Figure S2 Hepatic triglyceride levels show a trend to be decreased only 6 hours following UAG treatment.
(0.09 MB TIF)

Table S1 GSEA pathway gene sets up-regulated by UAG in GHSR KO white adipose tissue. [Size, number of genes in gene set; ES, enrichment score; NES, normalized enrichment score; NOM p-val, nominal p-value; FDR q-val, false detection rate q-value].
(0.05 MB DOC)

Table S2 GSEA pathway gene sets down-regulated by UAG in GHSR KO white adipose tissue. [Size, number of genes in gene set; ES, enrichment score; NES, normalized enrichment score; NOM p-val, nominal p-value; FDR q-val, false detection rate q-value].
(0.10 MB DOC)

Table S3 GSEA transcription factor target gene sets up-regulated by UAG in GHSR KO white adipose tissue. [Size, number of genes in gene set; ES, enrichment score; NES, normalized enrichment score; NOM p-val, nominal p-value; FDR q-val, false detection rate q-value].
(0.04 MB DOC)

Table S4 GSEA transcription factor target gene sets down-regulated by UAG in GHSR KO white adipose tissue. [Size, number of genes in gene set; ES, enrichment score; NES, normalized enrichment score; NOM p-val, nominal p-value; FDR q-val, false detection rate q-value].
(0.07 MB DOC)

Table S5 GSEA pathway gene sets up-regulated by UAG in GHSR KO muscle. [Size, number of genes in gene set; ES, enrichment score; NES, normalized enrichment score; NOM p-val, nominal p-value; FDR q-val, false detection rate q-value].
(0.06 MB DOC)

Table S6 GSEA pathway gene sets down-regulated by UAG in GHSR KO muscle. [Size, number of genes in gene set; ES, enrichment score; NES, normalized enrichment score; NOM p-val, nominal p-value; FDR q-val, false detection rate q-value].
(0.07 MB DOC)

Table S7 GSEA transcription factor target gene sets up-regulated by UAG in GHSR KO muscle. [Size, number of genes in gene set; ES, enrichment score; NES, normalized enrichment score; NOM p-val, nominal p-value; FDR q-val, false detection rate q-value].
(0.04 MB DOC)

Table S8 GSEA pathway gene sets up-regulated by UAG in GHSR KO liver. [Size, number of genes in gene set; ES, enrichment score; NES, normalized enrichment score; NOM p-val, nominal p-value; FDR q-val, false detection rate q-value].
(0.13 MB DOC)

Table S9 GSEA transcription factor target gene sets up-regulated by UAG in GHSR KO liver. [Size, number of genes in gene set; ES, enrichment score; NES, normalized enrichment score; NOM p-val, nominal p-value; FDR q-val, false detection rate q-value].
(0.03 MB DOC)

## References

[1] Kojima M, Hosoda H, Date Y, Nakazato M, Matsuo H (1999). Ghrelin is a growth-hormone-releasing acylated peptide from stomach.. Nature.

[2] Gutierrez JA, Solenberg PJ, Perkins DR, Willency JA, Knierman MD (2008). Ghrelin octanoylation mediated by an orphan lipid transferase.. Proc Natl Acad Sci U S A.

[3] Yang J, Brown MS, Liang G, Grishin NV, Goldstein JL (2008). Identification of the Acyltransferase that Octanoylates Ghrelin, an Appetite-Stimulating Peptide Hormone.. Cell.

[4] van der Lely AJ, Tschop M, Heiman ML, Ghigo E (2004). Biological, physiological, pathophysiological, and pharmacological aspects of ghrelin.. Endocr Rev.

[5] Granata R, Settanni F, Biancone L, Trovato L, Nano R (2007). Acylated and unacylated ghrelin promote proliferation and inhibit apoptosis of pancreatic beta-cells and human islets: involvement of 3′,5′-cyclic adenosine monophosphate/protein kinase A, extracellular signal-regulated kinase 1/2, and phosphatidyl inositol 3-Kinase/Akt signaling.. Endocrinology.

[6] Granata R, Volante M, Settanni F, Gauna C, Ghe C (2010). Unacylated ghrelin and obestatin increase islet cell mass and prevent diabetes in streptozotocin-treated newborn rats.. J Mol Endocrinol.

[7] Togliatto G, Trombetta A, Dentelli P, Baragli A, Rosso A (2010). Unacylated Ghrelin Rescues Endothelial Progenitor Cell Function in Individuals with Type 2 Diabetes.. Diabetes.

[8] Gauna C, Meyler FM, Janssen JA, Delhanty PJ, Abribat T (2004). Administration of acylated ghrelin reduces insulin sensitivity, whereas the combination of acylated plus unacylated ghrelin strongly improves insulin sensitivity.. J Clin Endocrinol Metab.

[9] Broglio F, Prodam F, Riganti F, Gramaglia E, Benso A (2007). Unacylated ghrelin (UAG) enhances the early insulin response to meal, improves glucose metabolism and decrease free fatty acids levels in healthy volunteers.. 89th Endocrine Society Meeting.

[10] Zhang W, Chai B, Li JY, Wang H, Mulholland MW (2008). Effect of des-acyl ghrelin on adiposity and glucose metabolism.. Endocrinology.

[11] Thompson NM, Gill DA, Davies R, Loveridge N, Houston PA (2004). Ghrelin and des-octanoyl ghrelin promote adipogenesis directly in vivo by a mechanism independent of GHS-R1a.. Endocrinology.

[12] Muccioli G, Pons N, Ghe C, Catapano F, Granata R (2004). Ghrelin and des-acyl ghrelin both inhibit isoproterenol-induced lipolysis in rat adipocytes via a non-type 1a growth hormone secretagogue receptor.. Eur J Pharmacol.

[13] Gauna C, Delhanty PJ, Hofland LJ, Janssen JA, Broglio F (2005). Ghrelin stimulates, whereas des-octanoyl ghrelin inhibits, glucose output by primary hepatocytes.. J Clin Endocrinol Metab.

[14] Heijboer AC, van den Hoek AM, Parlevliet ET, Havekes LM, Romijn JA (2006). Ghrelin differentially affects hepatic and peripheral insulin sensitivity in mice.. Diabetologia.

[15] Barazzoni R, Zanetti M, Ferreira C, Vinci P, Pirulli A (2007). Relationships between desacylated and acylated ghrelin and insulin sensitivity in the metabolic syndrome.. J Clin Endocrinol Metab.

[16] Liu J, Prudom CE, Nass R, Pezzoli SS, Oliveri MC (2008). Novel ghrelin assays provide evidence for independent regulation of ghrelin acylation and secretion in healthy young men.. J Clin Endocrinol Metab.

[17] Sun Y, Wang P, Zheng H, Smith RG (2004). Ghrelin stimulation of growth hormone release and appetite is mediated through the growth hormone secretagogue receptor.. Proc Natl Acad Sci U S A.

[18] Asakawa A, Inui A, Fujimiya M, Sakamaki R, Shinfuku N (2005). Stomach regulates energy balance via acylated ghrelin and desacyl ghrelin.. Gut.

[19] Subramanian A, Tamayo P, Mootha VK, Mukherjee S, Ebert BL (2005). Gene set enrichment analysis: a knowledge-based approach for interpreting genome-wide expression profiles.. Proc Natl Acad Sci U S A.

[20] Sekiya M, Yahagi N, Matsuzaka T, Takeuchi Y, Nakagawa Y (2007). SREBP-1-independent regulation of lipogenic gene expression in adipocytes.. J Lipid Res.

[21] Palmer DG, Rutter GA, Tavare JM (2002). Insulin-stimulated fatty acid synthase gene expression does not require increased sterol response element binding protein 1 transcription in primary adipocytes.. Biochem Biophys Res Commun.

[22] Horton JD, Goldstein JL, Brown MS (2002). SREBPs: activators of the complete program of cholesterol and fatty acid synthesis in the liver.. J Clin Invest.

[23] Farmer SR (2006). Transcriptional control of adipocyte formation.. Cell Metab.

[24] Ross DA, Hannenhalli S, Tobias JW, Cooch N, Shiekhattar R (2006). Functional analysis of Hes-1 in preadipocytes.. Mol Endocrinol.

[25] Derosa G, Ferrari I, D'Angelo A, Tinelli C, Salvadeo SA (2008). Matrix metalloproteinase−2 and −9 levels in obese patients.. Endothelium.

[26] Li Y, Wang Z, Furukawa N, Escaron P, Weiszmann J (2008). T2384, a novel antidiabetic agent with unique peroxisome proliferator-activated receptor gamma binding properties.. J Biol Chem.

[27] Yamauchi T, Kamon J, Waki H, Murakami K, Motojima K (2001). The mechanisms by which both heterozygous peroxisome proliferator-activated receptor gamma (PPARgamma) deficiency and PPARgamma agonist improve insulin resistance.. J Biol Chem.

[28] Gimpl G, Fahrenholz F (2001). The oxytocin receptor system: structure, function, and regulation.. Physiol Rev.

[29] Florian M, Jankowski M, Gutkowska J (2010). Oxytocin increases glucose uptake in neonatal rat cardiomyocytes.. Endocrinology.

[30] Lee ES, Uhm KO, Lee YM, Kwon J, Park SH (2008). Oxytocin stimulates glucose uptake in skeletal muscle cells through the calcium-CaMKK-AMPK pathway.. Regul Pept.

[31] Qiu G, Hill JS (2009). Endothelial lipase promotes apolipoprotein AI-mediated cholesterol efflux in THP-1 macrophages.. Arterioscler Thromb Vasc Biol.

[32] Lamarche B, Paradis ME (2007). Endothelial lipase and the metabolic syndrome.. Curr Opin Lipidol.

[33] Lafontan M, Langin D (2009). Lipolysis and lipid mobilization in human adipose tissue.. Prog Lipid Res.

[34] Itoh N, Sakaue S, Nakagawa H, Kurogochi M, Ohira H (2007). Analysis of N-glycan in serum glycoproteins from db/db mice and humans with type 2 diabetes.. Am J Physiol Endocrinol Metab.

[35] Berggreen C, Gormand A, Omar B, Degerman E, Goransson O (2009). Protein kinase B activity is required for the effects of insulin on lipid metabolism in adipocytes.. Am J Physiol Endocrinol Metab.

[36] Zhang J, Wu Y, Zhang Y, Leroith D, Bernlohr DA (2008). The role of lipocalin 2 in the regulation of inflammation in adipocytes and macrophages.. Mol Endocrinol.

[37] Yan QW, Yang Q, Mody N, Graham TE, Hsu CH (2007). The adipokine lipocalin 2 is regulated by obesity and promotes insulin resistance.. Diabetes.

[38] De Souza CT, Araujo EP, Stoppiglia LF, Pauli JR, Ropelle E (2007). Inhibition of UCP2 expression reverses diet-induced diabetes mellitus by effects on both insulin secretion and action.. Faseb J.

[39] Scroyen I, Jacobs F, Cosemans L, De Geest B, Lijnen HR (2009). Effect of plasminogen activator inhibitor-1 on adipogenesis in vivo.. Thromb Haemost.

[40] Venugopal J, Hanashiro K, Nagamine Y (2007). Regulation of PAI-1 gene expression during adipogenesis.. J Cell Biochem.

[41] Barazzoni R, Bosutti A, Stebel M, Cattin MR, Roder E (2005). Ghrelin Regulates Mitochondrial-Lipid Metabolism Gene Expression and Tissue Fat Distribution Favoring Triglyceride Deposition in Liver but Not Skeletal Muscle.. Am J Physiol Endocrinol Metab.

[42] Davies JS, Kotokorpi P, Eccles SR, Barnes SK, Tokarczuk PF (2009). Ghrelin induces abdominal obesity via GHS-R-dependent lipid retention.. Mol Endocrinol.

[43] Lavery GG, Walker EA, Turan N, Rogoff D, Ryder JW (2008). Deletion of hexose-6-phosphate dehydrogenase activates the unfolded protein response pathway and induces skeletal myopathy.. J Biol Chem.

[44] Inohara N, Koseki T, Chen S, Wu X, Nunez G (1998). CIDE, a novel family of cell death activators with homology to the 45 kDa subunit of the DNA fragmentation factor.. Embo J.

[45] Puri V, Ranjit S, Konda S, Nicoloro SM, Straubhaar J (2008). Cidea is associated with lipid droplets and insulin sensitivity in humans.. Proc Natl Acad Sci U S A.

[46] Zhou Z, Yon Toh S, Chen Z, Guo K, Ng CP (2003). Cidea-deficient mice have lean phenotype and are resistant to obesity.. Nat Genet.

[47] Aguiari P, Leo S, Zavan B, Vindigni V, Rimessi A (2008). High glucose induces adipogenic differentiation of muscle-derived stem cells.. Proc Natl Acad Sci U S A.

[48] Gerin I, Bommer GT, Lidell ME, Cederberg A, Enerback S (2009). On the Role of FOX Transcription Factors in Adipocyte Differentiation and Insulin-stimulated Glucose Uptake.. J Biol Chem.

[49] Greco AV, Mingrone G, Giancaterini A, Manco M, Morroni M (2002). Insulin resistance in morbid obesity: reversal with intramyocellular fat depletion.. Diabetes.

[50] Herrema H, Derks TG, van Dijk TH, Bloks VW, Gerding A (2008). Disturbed hepatic carbohydrate management during high metabolic demand in medium-chain acyl-CoA dehydrogenase (MCAD)-deficient mice.. Hepatology.

[51] Toshinai K, Yamaguchi H, Sun Y, Smith RG, Yamanaka A (2006). Des-acyl Ghrelin Induces Food Intake by a Mechanism Independent of the Growth Hormone Secretagogue Receptor.. Endocrinology.

[52] Inhoff T, Monnikes H, Noetzel S, Stengel A, Goebel M (2008). Desacyl ghrelin inhibits the orexigenic effect of peripherally injected ghrelin in rats.. Peptides.

[53] Theander-Carrillo C, Wiedmer P, Cettour-Rose P, Nogueiras R, Perez-Tilve D (2006). Ghrelin action in the brain controls adipocyte metabolism.. J Clin Invest.

[54] Sangiao-Alvarellos S, Vazquez MJ, Varela L, Nogueiras R, Saha AK (2009). Central ghrelin regulates peripheral lipid metabolism in a growth hormone-independent fashion.. Endocrinology.

[55] Andrews ZB, Erion DM, Beiler R, Choi CS, Shulman GI (2010). Uncoupling protein-2 decreases the lipogenic actions of ghrelin.. Endocrinology.

[56] Mano-Otagiri A, Ohata H, Iwasaki-Sekino A, Nemoto T, Shibasaki T (2009). Ghrelin suppresses noradrenaline release in the brown adipose tissue of rats.. J Endocrinol.

[57] Schroeder A, Mueller O, Stocker S, Salowsky R, Leiber M (2006). The RIN: an RNA integrity number for assigning integrity values to RNA measurements.. BMC Mol Biol.

