# Supplementary figures and images for: Unacylated Ghrelin Rapidly Modulates Lipogenic and Insulin Signaling Pathway Gene Expression in Metabolically Active Tissues of GHSR Deleted Mice
Source: PLoS One. 2010 Jul 26;5(7):e11749. doi: 10.1371/journal.pone.0011749 (PMC2909919; doi:10.1371/journal.pone.0011749)

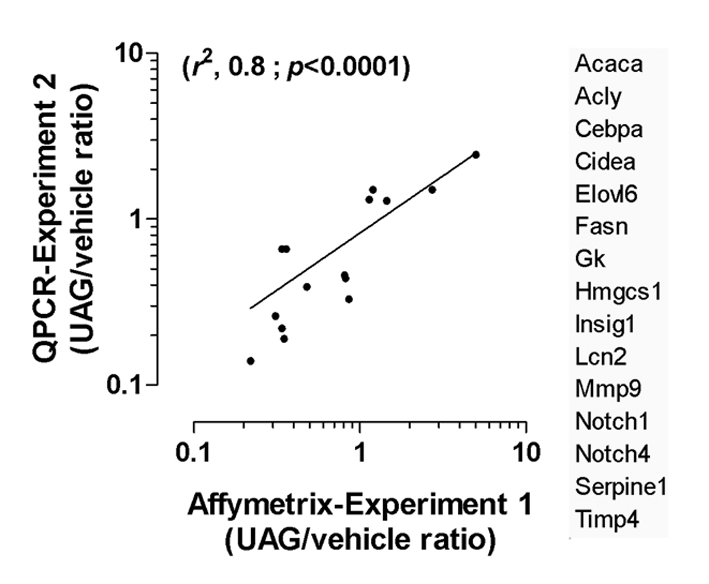

Supplement: Figure S1 — Approximately 70% of genes assessed by QPCR in fat from the independent Experiment 2 correlated strongly, in terms of direction and magnitude of regulation by UAG, with the array data derived from Experiment 1 (r2, 0.8; p<0.0001). (0.07 MB TIF) [file pone.0011749.s001.tif]
