# Supplementary material for: Unacylated Ghrelin Rapidly Modulates Lipogenic and Insulin Signaling Pathway Gene Expression in Metabolically Active Tissues of GHSR Deleted Mice
Source: PLoS One. 2010 Jul 26;5(7):e11749. doi: 10.1371/journal.pone.0011749 (PMC2909919; doi:10.1371/journal.pone.0011749)
Supplement: Table S1 — GSEA pathway gene sets up-regulated by UAG in GHSR KO white adipose tissue. [Size, number of genes in gene set; ES, enrichment score; NES, normalized enrichment score; NOM p-val, nominal p-value; FDR q-val, false detection rate q-value]. (0.05 MB DOC) [file pone.0011749.s003.doc]

| **NAME – Up-regulated in KO WAT** | **SIZE** | **ES** | **NES** | **NOM p-val** | **FDR q-val** |
| --- | --- | --- | --- | --- | --- |
| HSA00511_N_GLYCAN_DEGRADATION | 13 | 0.724 | 1.971 | 0.000 | 0.000 |
| PARKINPATHWAY | 10 | 0.532 | 1.864 | 0.000 | 0.000 |
| FIBRINOLYSISPATHWAY | 11 | 0.706 | 1.726 | 0.000 | 0.000 |
| CD40PATHWAY | 13 | 0.655 | 1.719 | 0.000 | 0.000 |
| N_GLYCAN_DEGRADATION | 10 | 0.731 | 1.704 | 0.000 | 0.020 |
| COMPPATHWAY | 12 | 0.695 | 1.643 | 0.000 | 0.048 |
| HSA04610_COMPLEMENT_AND_COAGULATION_CASCADES | 56 | 0.518 | 1.601 | 0.000 | 0.126 |
| AKTPATHWAY | 15 | 0.618 | 1.527 | 0.000 | 0.159 |
| HSA00480_GLUTATHIONE_METABOLISM | 30 | 0.474 | 1.513 | 0.000 | 0.216 |
| HSA05050_DENTATORUBROPALLIDOLUYSIAN_ATROPHY | 13 | 0.555 | 1.442 | 0.000 | 0.475 |
| TNFR2PATHWAY | 17 | 0.570 | 1.439 | 0.000 | 0.449 |
| IGF1MTORPATHWAY | 19 | 0.499 | 1.421 | 0.000 | 0.499 |
| COMPLEMENT_ACTIVATION_CLASSICAL | 10 | 0.719 | 1.418 | 0.000 | 0.476 |
| HSA00534_HEPARAN_SULFATE_BIOSYNTHESIS | 16 | 0.635 | 1.406 | 0.000 | 0.469 |
| HSA04710_CIRCADIAN_RHYTHM | 12 | 0.654 | 1.383 | 0.000 | 0.515 |
| HSA04330_NOTCH_SIGNALING_PATHWAY | 40 | 0.388 | 1.376 | 0.000 | 0.536 |
| O_GLYCAN_BIOSYNTHESIS | 10 | 0.543 | 1.338 | 0.000 | 0.727 |
| ST_WNT_CA2_CYCLIC_GMP_PATHWAY | 16 | 0.585 | 1.326 | 0.000 | 0.746 |
| P27PATHWAY | 12 | 0.472 | 1.311 | 0.000 | 0.764 |
| GPCRDB_OTHER | 31 | 0.326 | 1.300 | 0.000 | 0.796 |
