# Supplementary material for: Unacylated Ghrelin Rapidly Modulates Lipogenic and Insulin Signaling Pathway Gene Expression in Metabolically Active Tissues of GHSR Deleted Mice
Source: PLoS One. 2010 Jul 26;5(7):e11749. doi: 10.1371/journal.pone.0011749 (PMC2909919; doi:10.1371/journal.pone.0011749)
Supplement: Table S2 — GSEA pathway gene sets down-regulated by UAG in GHSR KO white adipose tissue. [Size, number of genes in gene set; ES, enrichment score; NES, normalized enrichment score; NOM p-val, nominal p-value; FDR q-val, false detection rate q-value]. (0.10 MB DOC) [file pone.0011749.s004.doc]

| **NAME – Down-regulated in KO WAT by UAG** | **SIZE** | **ES** | **NES** | **NOM p-val** | **FDR q-val** |
| --- | --- | --- | --- | --- | --- |
| CHOLESTEROL_BIOSYNTHESIS | 15 | -0.849 | -2.109 | 0.000 | 0.000 |
| HSA00010_GLYCOLYSIS_AND_GLUCONEOGENESIS | 48 | -0.678 | -2.055 | 0.000 | 0.000 |
| HSA00100_BIOSYNTHESIS_OF_STEROIDS | 22 | -0.798 | -1.928 | 0.000 | 0.015 |
| HSA00903_LIMONENE_AND_PINENE_DEGRADATION | 24 | -0.716 | -1.907 | 0.000 | 0.013 |
| HSA00190_OXIDATIVE_PHOSPHORYLATION | 101 | -0.560 | -1.893 | 0.000 | 0.012 |
| HSA00620_PYRUVATE_METABOLISM | 35 | -0.671 | -1.886 | 0.000 | 0.009 |
| HSA00521_STREPTOMYCIN_BIOSYNTHESIS | 10 | -0.747 | -1.870 | 0.000 | 0.009 |
| BIOSYNTHESIS_OF_STEROIDS | 13 | -0.828 | -1.856 | 0.000 | 0.016 |
| HSA00071_FATTY_ACID_METABOLISM | 40 | -0.664 | -1.855 | 0.000 | 0.015 |
| HSA04930_TYPE_II_DIABETES_MELLITUS | 38 | -0.608 | -1.809 | 0.000 | 0.020 |
| CDK5PATHWAY | 11 | -0.732 | -1.791 | 0.000 | 0.018 |
| MTA3PATHWAY | 13 | -0.660 | -1.780 | 0.000 | 0.017 |
| HSA00632_BENZOATE_DEGRADATION_VIA_COA_LIGATION | 25 | -0.676 | -1.776 | 0.000 | 0.022 |
| UBIQUINONE_BIOSYNTHESIS | 14 | -0.749 | -1.767 | 0.000 | 0.021 |
| ARGININE_AND_PROLINE_METABOLISM | 36 | -0.624 | -1.757 | 0.000 | 0.024 |
| HSA00624_1_AND_2_METHYLNAPHTHALENE_DEGRADATION | 20 | -0.582 | -1.746 | 0.000 | 0.027 |
| HSA00640_PROPANOATE_METABOLISM | 30 | -0.627 | -1.731 | 0.000 | 0.035 |
| ANDROGEN_AND_ESTROGEN_METABOLISM | 12 | -0.763 | -1.714 | 0.000 | 0.038 |
| HSA04020_CALCIUM_SIGNALING_PATHWAY | 135 | -0.526 | -1.713 | 0.000 | 0.036 |
| HSA00720_REDUCTIVE_CARBOXYLATE_CYCLE | 10 | -0.767 | -1.684 | 0.000 | 0.050 |
| PYRUVATE_METABOLISM | 31 | -0.536 | -1.684 | 0.000 | 0.048 |
| MITOCHONDRIAL_FATTY_ACID_BETAOXIDATION | 14 | -0.747 | -1.672 | 0.000 | 0.050 |
| PROPANOATE_METABOLISM | 29 | -0.550 | -1.661 | 0.000 | 0.046 |
| HSA04910_INSULIN_SIGNALING_PATHWAY | 123 | -0.456 | -1.651 | 0.000 | 0.045 |
| ECMPATHWAY | 21 | -0.601 | -1.635 | 0.000 | 0.050 |
| MCALPAINPATHWAY | 23 | -0.564 | -1.629 | 0.000 | 0.051 |
| HSA01040_POLYUNSATURATED_FATTY_ACID_BIOSYNTHESIS | 13 | -0.777 | -1.626 | 0.000 | 0.053 |
| HSA01430_CELL_COMMUNICATION | 59 | -0.478 | -1.626 | 0.000 | 0.054 |
| RAC1PATHWAY | 21 | -0.617 | -1.624 | 0.000 | 0.053 |
| FRUCTOSE_AND_MANNOSE_METABOLISM | 23 | -0.489 | -1.619 | 0.000 | 0.054 |
| MONOCYTEPATHWAY | 10 | -0.702 | -1.616 | 0.000 | 0.055 |
| P53HYPOXIAPATHWAY | 18 | -0.606 | -1.607 | 0.000 | 0.062 |
| OXIDATIVE_PHOSPHORYLATION | 53 | -0.545 | -1.606 | 0.000 | 0.060 |
| CCR3PATHWAY | 20 | -0.578 | -1.603 | 0.000 | 0.059 |
| HSA03010_RIBOSOME | 55 | -0.568 | -1.563 | 0.000 | 0.082 |
| UCALPAINPATHWAY | 15 | -0.742 | -1.561 | 0.000 | 0.080 |
| SMOOTH_MUSCLE_CONTRACTION | 131 | -0.460 | -1.559 | 0.000 | 0.080 |
| HSA04530_TIGHT_JUNCTION | 109 | -0.466 | -1.552 | 0.000 | 0.085 |
| CELL2CELLPATHWAY | 12 | -0.775 | -1.550 | 0.000 | 0.083 |
| HSA00710_CARBON_FIXATION | 19 | -0.634 | -1.547 | 0.000 | 0.086 |
| HSA00642_ETHYLBENZENE_DEGRADATION | 13 | -0.633 | -1.527 | 0.000 | 0.101 |
| HSA04512_ECM_RECEPTOR_INTERACTION | 70 | -0.517 | -1.518 | 0.000 | 0.104 |
| CALCIUM_REGULATION_IN_CARDIAC_CELLS | 124 | -0.438 | -1.510 | 0.000 | 0.106 |
| HSA04950_MATURITY_ONSET_DIABETES_OF_THE_YOUNG | 13 | -0.672 | -1.488 | 0.000 | 0.129 |
| INTEGRINPATHWAY | 34 | -0.609 | -1.484 | 0.000 | 0.134 |
| HSA00120_BILE_ACID_BIOSYNTHESIS | 29 | -0.621 | -1.482 | 0.000 | 0.134 |
| HSA04740_OLFACTORY_TRANSDUCTION | 22 | -0.519 | -1.469 | 0.000 | 0.140 |
| KREBS_TCA_CYCLE | 26 | -0.764 | -1.458 | 0.000 | 0.150 |
| HSA05130_PATHOGENIC_ESCHERICHIA_COLI_INFECTION_EHEC | 37 | -0.485 | -1.457 | 0.000 | 0.149 |
| CARBON_FIXATION | 18 | -0.620 | -1.455 | 0.000 | 0.147 |
| ACE2PATHWAY | 11 | -0.637 | -1.454 | 0.000 | 0.148 |
| HSA00500_STARCH_AND_SUCROSE_METABOLISM | 56 | -0.430 | -1.445 | 0.000 | 0.152 |
| HSA04510_FOCAL_ADHESION | 174 | -0.450 | -1.427 | 0.000 | 0.179 |
| LIMONENE_AND_PINENE_DEGRADATION | 11 | -0.620 | -1.426 | 0.000 | 0.176 |
| HSA00252_ALANINE_AND_ASPARTATE_METABOLISM | 29 | -0.492 | -1.406 | 0.000 | 0.202 |
| HSA03320_PPAR_SIGNALING_PATHWAY | 59 | -0.508 | -1.395 | 0.000 | 0.222 |
| HSA00562_INOSITOL_PHOSPHATE_METABOLISM | 42 | -0.489 | -1.383 | 0.000 | 0.233 |
