# Supplementary material for: Unacylated Ghrelin Rapidly Modulates Lipogenic and Insulin Signaling Pathway Gene Expression in Metabolically Active Tissues of GHSR Deleted Mice
Source: PLoS One. 2010 Jul 26;5(7):e11749. doi: 10.1371/journal.pone.0011749 (PMC2909919; doi:10.1371/journal.pone.0011749)
Supplement: Table S3 — GSEA transcription factor target gene sets up-regulated by UAG in GHSR KO white adipose tissue. [Size, number of genes in gene set; ES, enrichment score; NES, normalized enrichment score; NOM p-val, nominal p-value; FDR q-val, false detection rate q-value]. (0.04 MB DOC) [file pone.0011749.s005.doc]

| **NAME – TFTs Up-regulated in KO WAT** | **SIZE** | **ES** | **NES** | **NOM p-val** | **FDR q-val** |
| --- | --- | --- | --- | --- | --- |
| V$E2F4DP2_01 | 151 | 0.377 | 1.704 | 0.000 | 0.000 |
| YGCANTGCR_UNKNOWN | 90 | 0.367 | 1.638 | 0.000 | 0.028 |
| RYAAAKNNNNNNTTGW_UNKNOWN | 54 | 0.476 | 1.512 | 0.000 | 0.034 |
| V$CREBP1_01 | 115 | 0.388 | 1.432 | 0.000 | 0.089 |
| V$E2F1DP2_01 | 151 | 0.377 | 1.418 | 0.000 | 0.091 |
| CYTAGCAAY_UNKNOWN | 88 | 0.342 | 1.399 | 0.000 | 0.076 |
| V$E2F_02 | 151 | 0.371 | 1.396 | 0.000 | 0.065 |
| V$E2F1DP1_01 | 151 | 0.377 | 1.371 | 0.000 | 0.076 |
| RYTAAWNNNTGAY_UNKNOWN | 44 | 0.428 | 1.361 | 0.000 | 0.078 |
| SGCGSSAAA_V$E2F1DP2_01 | 112 | 0.345 | 1.325 | 0.000 | 0.115 |
| TTGCWCAAY_V$CEBPB_02 | 47 | 0.349 | 1.280 | 0.200 | 0.192 |
| V$NRSF_01 | 41 | 0.408 | 1.272 | 0.000 | 0.202 |
| YGACNNYACAR_UNKNOWN | 53 | 0.358 | 1.258 | 0.000 | 0.213 |
| V$E2F_03 | 162 | 0.301 | 1.248 | 0.000 | 0.237 |
