# Supplementary material for: Unacylated Ghrelin Rapidly Modulates Lipogenic and Insulin Signaling Pathway Gene Expression in Metabolically Active Tissues of GHSR Deleted Mice
Source: PLoS One. 2010 Jul 26;5(7):e11749. doi: 10.1371/journal.pone.0011749 (PMC2909919; doi:10.1371/journal.pone.0011749)
Supplement: Table S4 — GSEA transcription factor target gene sets down-regulated by UAG in GHSR KO white adipose tissue. [Size, number of genes in gene set; ES, enrichment score; NES, normalized enrichment score; NOM p-val, nominal p-value; FDR q-val, false detection rate q-value]. (0.07 MB DOC) [file pone.0011749.s006.doc]

| **NAME – TFTs Down-regulated in KO WAT (bottom 35)** | **SIZE** | **ES** | **NES** | **NOM p-val** | **FDR q-val** |
| --- | --- | --- | --- | --- | --- |
| V$SRF_Q5_01 | 165 | -0.624 | -2.197 | 0.000 | 0.000 |
| CCAWWNAAGG_V$SRF_Q4 | 65 | -0.693 | -2.083 | 0.000 | 0.000 |
| V$SRF_Q6 | 181 | -0.568 | -2.019 | 0.000 | 0.000 |
| V$SRF_Q4 | 161 | -0.602 | -1.985 | 0.000 | 0.000 |
| V$TATA_01 | 155 | -0.594 | -1.956 | 0.000 | 0.000 |
| WWTAAGGC_UNKNOWN | 99 | -0.623 | -1.956 | 0.000 | 0.000 |
| V$PAX2_01 | 29 | -0.714 | -1.868 | 0.000 | 0.000 |
| V$HEB_Q6 | 158 | -0.541 | -1.862 | 0.000 | 0.000 |
| V$ER_Q6 | 171 | -0.468 | -1.827 | 0.000 | 0.000 |
| RACTNNRTTTNC_UNKNOWN | 71 | -0.481 | -1.788 | 0.000 | 0.000 |
| V$MEF2_02 | 153 | -0.526 | -1.776 | 0.000 | 0.000 |
| V$T3R_Q6 | 157 | -0.474 | -1.754 | 0.000 | 0.000 |
| V$GFI1_01 | 175 | -0.494 | -1.743 | 0.000 | 0.000 |
| V$HMEF2_Q6 | 88 | -0.537 | -1.717 | 0.000 | 0.000 |
| V$SF1_Q6 | 160 | -0.499 | -1.716 | 0.000 | 0.000 |
| YTATTTTNR_V$MEF2_02 | 449 | -0.416 | -1.704 | 0.000 | 0.000 |
| V$EVI1_04 | 146 | -0.491 | -1.680 | 0.000 | 0.000 |
| V$TBP_01 | 157 | -0.508 | -1.653 | 0.000 | 0.000 |
| V$EVI1_02 | 76 | -0.485 | -1.652 | 0.000 | 0.000 |
| V$MEF2_Q6_01 | 167 | -0.497 | -1.644 | 0.000 | 0.000 |
| V$TAL1BETAITF2_01 | 164 | -0.448 | -1.635 | 0.000 | 0.000 |
| V$P53_02 | 159 | -0.484 | -1.634 | 0.000 | 0.000 |
| CTAWWWATA_V$RSRFC4_Q2 | 249 | -0.502 | -1.628 | 0.000 | 0.005 |
| V$PAX8_01 | 24 | -0.675 | -1.611 | 0.000 | 0.019 |
| TGACCTTG_V$SF1_Q6 | 156 | -0.457 | -1.610 | 0.000 | 0.018 |
| V$CEBP_Q3 | 163 | -0.486 | -1.603 | 0.000 | 0.018 |
| V$MMEF2_Q6 | 181 | -0.433 | -1.596 | 0.000 | 0.017 |
| V$PAX5_01 | 96 | -0.401 | -1.582 | 0.000 | 0.021 |
| V$TAL1BETAE47_01 | 152 | -0.458 | -1.580 | 0.000 | 0.020 |
| V$TFIIA_Q6 | 173 | -0.469 | -1.579 | 0.000 | 0.019 |
| V$HFH4_01 | 137 | -0.452 | -1.572 | 0.000 | 0.023 |
| V$FOXD3_01 | 141 | -0.479 | -1.570 | 0.000 | 0.026 |
| V$NKX3A_01 | 135 | -0.468 | -1.568 | 0.000 | 0.029 |
| V$HFH8_01 | 148 | -0.457 | -1.564 | 0.000 | 0.028 |
| V$AP2REP_01 | 114 | -0.442 | -1.552 | 0.000 | 0.027 |
