# Supplementary material for: Unacylated Ghrelin Rapidly Modulates Lipogenic and Insulin Signaling Pathway Gene Expression in Metabolically Active Tissues of GHSR Deleted Mice
Source: PLoS One. 2010 Jul 26;5(7):e11749. doi: 10.1371/journal.pone.0011749 (PMC2909919; doi:10.1371/journal.pone.0011749)
Supplement: Table S5 — GSEA pathway gene sets up-regulated by UAG in GHSR KO muscle. [Size, number of genes in gene set; ES, enrichment score; NES, normalized enrichment score; NOM p-val, nominal p-value; FDR q-val, false detection rate q-value]. (0.06 MB DOC) [file pone.0011749.s007.doc]

| **NAME – Up-regulated in KO Muscle by UAG** | **SIZE** | **ES** | **NES** | **NOM p-val** | **FDR q-val** |
| --- | --- | --- | --- | --- | --- |
| CARIES_PULP_HIGH_UP | 69 | 0.751 | 2.659 | 0.000 | 0.000 |
| CARIES_PULP_UP | 162 | 0.675 | 2.583 | 0.000 | 0.000 |
| LIAN_MYELOID_DIFF_GRANULE | 25 | 0.834 | 2.557 | 0.000 | 0.000 |
| HOHENKIRK_MONOCYTE_DEND_DN | 97 | 0.668 | 2.496 | 0.000 | 0.000 |
| HADDAD_HSC_CD7_UP | 49 | 0.776 | 2.438 | 0.000 | 0.000 |
| HADDAD_CD45CD7_PLUS_VS_MINUS_UP | 49 | 0.776 | 2.409 | 0.000 | 0.000 |
| ROSS_CBF_MYH | 42 | 0.746 | 2.402 | 0.000 | 0.000 |
| PARK_RARALPHA_MOD | 51 | 0.685 | 2.352 | 0.000 | 0.000 |
| LAL_KO_3MO_UP | 41 | 0.808 | 2.273 | 0.000 | 0.000 |
| STEMCELL_COMMON_DN | 55 | 0.631 | 2.269 | 0.000 | 0.000 |
| NADLER_OBESITY_UP | 56 | 0.693 | 2.207 | 0.000 | 0.000 |
| BLOOD_CLOTTING_CASCADE | 19 | 0.800 | 2.182 | 0.000 | 0.000 |
| NI2_MOUSE_UP | 39 | 0.690 | 2.174 | 0.000 | 0.000 |
| IRITANI_ADPROX_VASC | 131 | 0.520 | 2.134 | 0.000 | 0.000 |
| TAVOR_CEBP_UP | 40 | 0.716 | 2.082 | 0.000 | 0.000 |
| MARTINELLI_IFNS_DIFF | 15 | 0.936 | 2.071 | 0.000 | 0.000 |
| ST_GRANULE_CELL_SURVIVAL_PATHWAY | 25 | 0.611 | 2.056 | 0.000 | 0.000 |
| HOFFMANN_BIVSBII_BI_TABLE2 | 221 | 0.466 | 2.031 | 0.000 | 0.000 |
| DNA_REPLICATION_REACTOME | 38 | 0.561 | 2.027 | 0.000 | 0.000 |
| FLECHNER_KIDNEY_TRANSPLANT_REJECTION_UP | 70 | 0.680 | 2.023 | 0.000 | 0.000 |
| GO_ROS | 25 | 0.710 | 2.021 | 0.000 | 0.000 |
| IDX_TSA_UP_CLUSTER3 | 82 | 0.585 | 2.019 | 0.000 | 0.000 |
| CROONQUIST_RAS_STROMA_DN | 19 | 0.673 | 2.014 | 0.000 | 0.000 |
| ZHAN_MMPC_SIMAL | 43 | 0.620 | 1.982 | 0.000 | 0.000 |
| CHIARETTI_T_ALL | 199 | 0.517 | 1.974 | 0.000 | 0.000 |
| ADIP_DIFF_CLUSTER2 | 38 | 0.678 | 1.969 | 0.000 | 0.000 |
| VEGF_MMMEC_12HRS_UP | 28 | 0.613 | 1.958 | 0.000 | 0.000 |
| IGF_VS_PDGF_DN | 39 | 0.616 | 1.955 | 0.000 | 0.004 |
| IDX_TSA_DN_CLUSTER2 | 60 | 0.533 | 1.938 | 0.000 | 0.004 |
| IDX_TSA_UP_CLUSTER1 | 24 | 0.777 | 1.936 | 0.000 | 0.004 |
| BOQUEST_CD31PLUS_VS_CD31MINUS_DN | 225 | 0.510 | 1.934 | 0.000 | 0.004 |
| LEE_DENA_UP | 50 | 0.656 | 1.931 | 0.000 | 0.004 |
| CROONQUIST_IL6_STARVE_UP | 32 | 0.711 | 1.928 | 0.000 | 0.004 |
| CHIARETTI_T_ALL_DIFF | 214 | 0.497 | 1.928 | 0.000 | 0.004 |
| SCHURINGA_STAT5A_DN | 16 | 0.836 | 1.894 | 0.000 | 0.003 |
