# Supplementary material for: Unacylated Ghrelin Rapidly Modulates Lipogenic and Insulin Signaling Pathway Gene Expression in Metabolically Active Tissues of GHSR Deleted Mice
Source: PLoS One. 2010 Jul 26;5(7):e11749. doi: 10.1371/journal.pone.0011749 (PMC2909919; doi:10.1371/journal.pone.0011749)
Supplement: Table S6 — GSEA pathway gene sets down-regulated by UAG in GHSR KO muscle. [Size, number of genes in gene set; ES, enrichment score; NES, normalized enrichment score; NOM p-val, nominal p-value; FDR q-val, false detection rate q-value]. (0.07 MB DOC) [file pone.0011749.s008.doc]

| **NAME – Down-regulated in KO Muscle by UAG** | **SIZE** | **ES** | **NES** | **NOM p-val** | **FDR q-val** |
| --- | --- | --- | --- | --- | --- |
| PENTOSE_PHOSPHATE_PATHWAY | 20 | -0.719 | -2.228 | 0.000 | 0.000 |
| HSA00030_PENTOSE_PHOSPHATE_PATHWAY | 22 | -0.738 | -2.068 | 0.000 | 0.000 |
| BECKER_TAMOXIFEN_RESISTANT_DN | 42 | -0.587 | -1.952 | 0.000 | 0.000 |
| HSA04740_OLFACTORY_TRANSDUCTION | 22 | -0.724 | -1.878 | 0.000 | 0.000 |
| IFNA_HCMV_6HRS_UP | 39 | -0.549 | -1.859 | 0.000 | 0.000 |
| ADIP_DIFF_UP | 66 | -0.573 | -1.834 | 0.000 | 0.000 |
| IDX_TSA_DN_CLUSTER6 | 21 | -0.638 | -1.808 | 0.000 | 0.000 |
| TNFALPHA_ADIP_DN | 57 | -0.604 | -1.786 | 0.000 | 0.000 |
| HSA00051_FRUCTOSE_AND_MANNOSE_METABOLISM | 37 | -0.570 | -1.738 | 0.000 | 0.023 |
| IFN_ALPHA_UP | 33 | -0.494 | -1.710 | 0.000 | 0.042 |
| TRYPTOPHAN_METABOLISM | 35 | -0.588 | -1.709 | 0.000 | 0.039 |
| TAKEDA_NUP8_HOXA9_3D_UP | 128 | -0.436 | -1.701 | 0.000 | 0.053 |
| CHOLESTEROL_BIOSYNTHESIS | 15 | -0.664 | -1.695 | 0.000 | 0.060 |
| HSA00020_CITRATE_CYCLE | 24 | -0.530 | -1.693 | 0.000 | 0.055 |
| TNFALPHA_TGZ_ADIP_DN | 27 | -0.626 | -1.667 | 0.000 | 0.071 |
| NADLER_OBESITY_DN | 35 | -0.518 | -1.666 | 0.000 | 0.066 |
| HSA00100_BIOSYNTHESIS_OF_STEROIDS | 22 | -0.529 | -1.662 | 0.000 | 0.066 |
| TAKEDA_NUP8_HOXA9_8D_UP | 103 | -0.426 | -1.632 | 0.000 | 0.090 |
| IL7PATHWAY | 16 | -0.551 | -1.631 | 0.000 | 0.085 |
| IFN_ALL_UP | 17 | -0.681 | -1.624 | 0.000 | 0.085 |
| FATTY_ACID_DEGRADATION | 22 | -0.575 | -1.621 | 0.000 | 0.084 |
| ADIP_VS_PREADIP_UP | 34 | -0.556 | -1.616 | 0.000 | 0.084 |
| UVB_NHEK3_C5 | 33 | -0.491 | -1.594 | 0.000 | 0.092 |
| TESTIS_EXPRESSED_GENES | 35 | -0.483 | -1.574 | 0.000 | 0.097 |
| CHESLER_HIGHEST_FOLD_RANGE_GENES | 44 | -0.444 | -1.562 | 0.000 | 0.102 |
| TGZ_ADIP_UP | 15 | -0.573 | -1.546 | 0.000 | 0.120 |
| TAKEDA_NUP8_HOXA9_16D_UP | 107 | -0.464 | -1.543 | 0.000 | 0.120 |
| GAMMA_ESR_OLD_UNREG | 21 | -0.489 | -1.524 | 0.000 | 0.139 |
| LEE_TCELLS6_UP | 16 | -0.634 | -1.522 | 0.000 | 0.134 |
| HSA00565_ETHER_LIPID_METABOLISM | 26 | -0.477 | -1.507 | 0.000 | 0.150 |
| TAKEDA_NUP8_HOXA9_10D_UP | 125 | -0.440 | -1.483 | 0.000 | 0.190 |
| HSA00380_TRYPTOPHAN_METABOLISM | 51 | -0.525 | -1.481 | 0.000 | 0.193 |
| HSA00280_VALINE_LEUCINE_AND_ISOLEUCINE_DEGRADATION | 41 | -0.491 | -1.454 | 0.000 | 0.238 |
