# Supplementary material for: Unacylated Ghrelin Rapidly Modulates Lipogenic and Insulin Signaling Pathway Gene Expression in Metabolically Active Tissues of GHSR Deleted Mice
Source: PLoS One. 2010 Jul 26;5(7):e11749. doi: 10.1371/journal.pone.0011749 (PMC2909919; doi:10.1371/journal.pone.0011749)
Supplement: Table S7 — GSEA transcription factor target gene sets up-regulated by UAG in GHSR KO muscle. [Size, number of genes in gene set; ES, enrichment score; NES, normalized enrichment score; NOM p-val, nominal p-value; FDR q-val, false detection rate q-value]. (0.04 MB DOC) [file pone.0011749.s009.doc]

| **NAME Up-regulated in KO Muscle** | **SIZE** | **ES** | **NES** | **NOM p-val** | **FDR q-val** |
| --- | --- | --- | --- | --- | --- |
| V$CEBPB_02 | 182 | 0.473 | 1.871 | 0.000 | 0.000 |
| V$TAXCREB_02 | 16 | 0.614 | 1.712 | 0.000 | 0.000 |
| V$E2F_01 | 49 | 0.488 | 1.607 | 0.000 | 0.015 |
| GATAAGR_V$GATA_C | 186 | 0.364 | 1.432 | 0.000 | 0.120 |
| V$MYOGENIN_Q6 | 150 | 0.398 | 1.408 | 0.000 | 0.139 |
| V$SREBP_Q3 | 153 | 0.364 | 1.392 | 0.000 | 0.154 |
| V$SREBP1_02 | 57 | 0.487 | 1.392 | 0.000 | 0.154 |
| V$E2F_Q3 | 148 | 0.382 | 1.370 | 0.000 | 0.160 |
| V$CEBP_Q2 | 151 | 0.373 | 1.344 | 0.000 | 0.200 |
| V$CEBPDELTA_Q6 | 165 | 0.410 | 1.334 | 0.000 | 0.207 |
| V$MYOD_Q6_01 | 143 | 0.351 | 1.332 | 0.000 | 0.199 |
| V$MYOD_01 | 147 | 0.352 | 1.311 | 0.000 | 0.225 |
| V$E2F_Q6_01 | 155 | 0.386 | 1.306 | 0.000 | 0.225 |
