# Supplementary material for: Unacylated Ghrelin Rapidly Modulates Lipogenic and Insulin Signaling Pathway Gene Expression in Metabolically Active Tissues of GHSR Deleted Mice
Source: PLoS One. 2010 Jul 26;5(7):e11749. doi: 10.1371/journal.pone.0011749 (PMC2909919; doi:10.1371/journal.pone.0011749)
Supplement: Table S8 — GSEA pathway gene sets up-regulated by UAG in GHSR KO liver. [Size, number of genes in gene set; ES, enrichment score; NES, normalized enrichment score; NOM p-val, nominal p-value; FDR q-val, false detection rate q-value]. (0.13 MB DOC) [file pone.0011749.s010.doc]

| **NAME – Up-regulated in KO Liver by UAG** | **SIZE** | **ES** | **NES** | **NOM p-val** | **FDR q-val** |
| --- | --- | --- | --- | --- | --- |
| IDX_TSA_UP_CLUSTER1 | 24 | 0.792 | 2.550 | 0.000 | 0.000 |
| LEE_MYC_E2F1_UP | 44 | 0.592 | 2.182 | 0.000 | 0.000 |
| ADIP_DIFF_CLUSTER2 | 38 | 0.733 | 2.176 | 0.000 | 0.000 |
| NI2_MOUSE_UP | 39 | 0.568 | 2.114 | 0.000 | 0.000 |
| NI2_LUNG_DN | 15 | 0.743 | 2.030 | 0.000 | 0.000 |
| HOUSTIS_ROS | 34 | 0.582 | 1.999 | 0.000 | 0.000 |
| RORIE_ES_PNET_UP | 21 | 0.673 | 1.968 | 0.000 | 0.000 |
| UVB_NHEK3_C8 | 60 | 0.500 | 1.915 | 0.000 | 0.000 |
| VEGF_MMMEC_6HRS_UP | 46 | 0.547 | 1.884 | 0.000 | 0.000 |
| LEE_MYC_DN | 48 | 0.466 | 1.826 | 0.000 | 0.000 |
| CROONQUIST_RAS_STROMA_DN | 19 | 0.579 | 1.817 | 0.000 | 0.012 |
| GALINDO_ACT_UP | 74 | 0.554 | 1.792 | 0.000 | 0.011 |
| HSA01031_GLYCAN_STRUCTURES_BIOSYNTHESIS_2 | 51 | 0.536 | 1.778 | 0.000 | 0.010 |
| LVAD_HEARTFAILURE_UP | 77 | 0.555 | 1.760 | 0.000 | 0.019 |
| CARDIACEGFPATHWAY | 17 | 0.613 | 1.751 | 0.000 | 0.017 |
| ZHAN_MM_MOLECULAR_CLASSI_UP | 50 | 0.550 | 1.746 | 0.000 | 0.016 |
| LEE_MYC_UP | 50 | 0.514 | 1.743 | 0.000 | 0.015 |
| DORSAM_HOXA9_DN | 25 | 0.724 | 1.737 | 0.000 | 0.014 |
| CROONQUIST_IL6_STROMA_UP | 34 | 0.627 | 1.732 | 0.000 | 0.014 |
| ADIPOGENESIS_HMSC_CLASS3_UP | 59 | 0.453 | 1.704 | 0.000 | 0.019 |
| ZHAN_MM_CD138_HP_VS_REST | 32 | 0.597 | 1.691 | 0.000 | 0.031 |
| AGED_MOUSE_HIPPOCAMPUS_ANY_UP | 41 | 0.462 | 1.686 | 0.000 | 0.035 |
| ADDYA_K562_HEMIN_TREATMENT | 59 | 0.545 | 1.673 | 0.000 | 0.045 |
| BRG1_ALAB_DN | 31 | 0.511 | 1.660 | 0.000 | 0.048 |
| IGF1_NIH3T3_UP | 33 | 0.517 | 1.654 | 0.000 | 0.052 |
| HSA04740_OLFACTORY_TRANSDUCTION | 22 | 0.636 | 1.652 | 0.000 | 0.050 |
| HSA00604_GLYCOSPHINGOLIPID_BIOSYNTHESIS_GANGLIOSERIES | 15 | 0.679 | 1.649 | 0.000 | 0.048 |
| CROONQUIST_IL6_RAS_UP | 18 | 0.552 | 1.649 | 0.000 | 0.046 |
| CMV_HCMV_TIMECOURSE_20HRS_DN | 31 | 0.520 | 1.648 | 0.000 | 0.045 |
| ZHAN_MM_CD138_CD1_VS_REST | 35 | 0.506 | 1.629 | 0.000 | 0.073 |
| CDMACPATHWAY | 15 | 0.709 | 1.628 | 0.000 | 0.075 |
| UVB_NHEK3_C3 | 15 | 0.604 | 1.626 | 0.000 | 0.073 |
| LAL_KO_3MO_UP | 41 | 0.465 | 1.600 | 0.000 | 0.099 |
| ZMPSTE24_KO_DN | 25 | 0.586 | 1.599 | 0.000 | 0.096 |
| HYPERTROPHY_MODEL | 16 | 0.649 | 1.590 | 0.000 | 0.104 |
| IDX_TSA_UP_CLUSTER5 | 90 | 0.477 | 1.589 | 0.000 | 0.101 |
| PASSERINI_PROLIFERATION | 54 | 0.516 | 1.576 | 0.000 | 0.105 |
| ST_WNT_CA2_CYCLIC_GMP_PATHWAY | 16 | 0.661 | 1.573 | 0.000 | 0.106 |
| VERHAAK_AML_NPM1_MUT_VS_WT_UP | 131 | 0.396 | 1.564 | 0.000 | 0.110 |
| CAMPTOTHECIN_PROBCELL_UP | 21 | 0.512 | 1.542 | 0.000 | 0.141 |
| CREBPATHWAY | 26 | 0.531 | 1.540 | 0.000 | 0.144 |
| NAKAJIMA_MCS_UP | 74 | 0.438 | 1.540 | 0.000 | 0.144 |
| TIDPATHWAY | 15 | 0.541 | 1.538 | 0.000 | 0.143 |
| HSA00190_OXIDATIVE_PHOSPHORYLATION | 101 | 0.412 | 1.535 | 0.000 | 0.146 |
| BRCA1_SW480_UP | 23 | 0.477 | 1.529 | 0.000 | 0.154 |
| O6BG_RESIST_MEDULLOBLASTOMA_DN | 44 | 0.397 | 1.521 | 0.000 | 0.169 |
| UV_UNIQUE_FIBRO_UP | 18 | 0.635 | 1.518 | 0.000 | 0.168 |
| TNFR2PATHWAY | 17 | 0.555 | 1.513 | 0.000 | 0.170 |
| HDACI_COLON_TSA48HRS_UP | 28 | 0.573 | 1.512 | 0.000 | 0.167 |
| HSA00252_ALANINE_AND_ASPARTATE_METABOLISM | 29 | 0.524 | 1.506 | 0.000 | 0.171 |
| IL1_CORNEA_UP | 50 | 0.484 | 1.505 | 0.000 | 0.168 |
| HDACI_COLON_TSA2HRS_UP | 45 | 0.470 | 1.502 | 0.000 | 0.170 |
| CITRATE_CYCLE_TCA_CYCLE | 17 | 0.597 | 1.502 | 0.000 | 0.169 |
| HEARTFAILURE_ATRIA_UP | 22 | 0.499 | 1.497 | 0.000 | 0.177 |
| VEGF_MMMEC_12HRS_UP | 28 | 0.532 | 1.495 | 0.000 | 0.178 |
| PASSERINI_GROWTH | 31 | 0.454 | 1.493 | 0.000 | 0.182 |
| UV-CMV_UNIQUE_HCMV_6HRS_UP | 86 | 0.419 | 1.492 | 0.000 | 0.181 |
| TPA_RESIST_EARLY_DN | 62 | 0.504 | 1.489 | 0.000 | 0.180 |
| CHIARETTI_T_ALL | 199 | 0.366 | 1.488 | 0.000 | 0.183 |
| CMV_HCMV_TIMECOURSE_8HRS_UP | 16 | 0.476 | 1.487 | 0.000 | 0.180 |
| BRENTANI_TRANSPORT_OF_VESICLES | 22 | 0.507 | 1.480 | 0.000 | 0.183 |
| HDACI_COLON_BUT2HRS_UP | 55 | 0.397 | 1.472 | 0.000 | 0.187 |
| INFLAMMATORY_RESPONSE_PATHWAY | 20 | 0.538 | 1.467 | 0.000 | 0.199 |
| ELECTRON_TRANSPORT_CHAIN | 89 | 0.405 | 1.465 | 0.000 | 0.198 |
| PYRIMIDINE_METABOLISM | 58 | 0.451 | 1.462 | 0.000 | 0.199 |
| ST_GRANULE_CELL_SURVIVAL_PATHWAY | 25 | 0.534 | 1.461 | 0.000 | 0.196 |
| ST_MYOCYTE_AD_PATHWAY | 23 | 0.512 | 1.459 | 0.000 | 0.194 |
| WERNER_FIBRO_UP | 43 | 0.448 | 1.457 | 0.000 | 0.198 |
| CELL_GROWTH_AND_OR_MAINTENANCE | 55 | 0.517 | 1.454 | 0.000 | 0.199 |
| XU_ATRA_PLUSNSC_DN | 15 | 0.504 | 1.452 | 0.000 | 0.201 |
| TSA_HEPATOMA_UP | 33 | 0.563 | 1.443 | 0.000 | 0.213 |
| DAC_PANC50_UP | 30 | 0.610 | 1.433 | 0.000 | 0.236 |
| LIAN_MYELOID_DIFF_TF | 34 | 0.481 | 1.432 | 0.000 | 0.233 |
| UVB_NHEK1_UP | 136 | 0.379 | 1.429 | 0.000 | 0.236 |
| 41BBPATHWAY | 15 | 0.632 | 1.427 | 0.000 | 0.238 |
| MANALO_HYPOXIA_UP | 87 | 0.432 | 1.424 | 0.000 | 0.242 |
| KERATINOCYTEPATHWAY | 40 | 0.416 | 1.421 | 0.000 | 0.248 |
