# Supplementary material for: Unacylated Ghrelin Rapidly Modulates Lipogenic and Insulin Signaling Pathway Gene Expression in Metabolically Active Tissues of GHSR Deleted Mice
Source: PLoS One. 2010 Jul 26;5(7):e11749. doi: 10.1371/journal.pone.0011749 (PMC2909919; doi:10.1371/journal.pone.0011749)
Supplement: Table S9 — GSEA transcription factor target gene sets up-regulated by UAG in GHSR KO liver. [Size, number of genes in gene set; ES, enrichment score; NES, normalized enrichment score; NOM p-val, nominal p-value; FDR q-val, false detection rate q-value]. (0.03 MB DOC) [file pone.0011749.s011.doc]

| **NAME – TFTs upregulated in KO Liver** | **SIZE** | **ES** | **NES** | **NOM p-val** | **FDR q-val** |
| --- | --- | --- | --- | --- | --- |
| V$ATF_B | 122 | 0.415 | 1.597 | 0.000 | 0.100 |
| V$CREBP1_Q2 | 155 | 0.380 | 1.481 | 0.000 | 0.194 |
| V$RSRFC4_Q2 | 154 | 0.347 | 1.471 | 0.000 | 0.129 |
